# Genetic Characterization of Spring Wheat Germplasm for Macro-, Microelements and Trace Metals

**DOI:** 10.3390/plants11162173

**Published:** 2022-08-21

**Authors:** Alexey Morgounov, Huihui Li, Sergey Shepelev, Mohsin Ali, Paulina Flis, Hamit Koksel, Timur Savin, Vladimir Shamanin

**Affiliations:** 1 Agronomy Department, Omsk State Agrarian University, 644008 Omsk, Russia; 2 Institute of Crop Sciences, Chinese Academy of Agricultural Sciences & CIMMYT-China, Beijing 100081, China; 3 Nanfan Research Institute, Chinese Academy of Agricultural Sciences (CAAS), Sanya 572024, China; 4 Future Food Beacon of Excellence and the School of Biosciences, University of Nottingham, Nottingham LE12 5RD, UK; 5 Department of Nutrition and Dietetics, Istinye University, Istanbul 34010, Turkey; 6 Department of Research, S. Seifullin Kazakh Agro Technical University, Nur-Sultan 010011, Kazakhstan

**Keywords:** spring wheat, GWAS, macroelements, microelements, trace metals, functional genes

## Abstract

Wheat as a staple food crop is the main source of micro- and macronutrients for most people of the world and is recognized as an attractive crop for biofortification. This study presents a comprehensive investigation of genomic regions governing grain micro- and macroelements concentrations in a panel of 135 diverse wheat accessions through a genome-wide association study. The genetic diversity panel was genotyped using the genotyping-by-sequencing (GBS) method and phenotyped in two environments during 2017–2018. Wide ranges of variation in nutrient element concentrations in grain were detected among the accessions. Based on 33,808 high-quality single nucleotide polymorphisms (SNPs), 2997 marker-element associations (MEAs) with −log10(*p*-value) > 3.5 were identified, representing all three subgenomes of wheat for 15-grain concentration elements. The highest numbers of MEAs were identified for Mg (499), followed by S (399), P (394), Ni (381), Cd (243), Ca (229), Mn (224), Zn (212), Sr (212), Cu (111), Rb (78), Fe (63), Mo (43), K (32) and Co (19). Further, MEAs associated with multiple elements and referred to as pleiotropic SNPs were identified for Mg, P, Cd, Mn, and Zn on chromosomes 1B, 2B, and 6B. Fifty MEAs were subjected to validation using KASIB multilocational trial at six sites in two years using 39 genotypes. Gene annotation of MEAs identified putative candidate genes that potentially encode different types of proteins related to disease, metal transportation, and metabolism. The MEAs identified in the present study could be potential targets for further validation and may be used in marker-assisted breeding to improve nutrient element concentrations in wheat grain.

## 1. Introduction

Spring wheat is an important crop in Kazakhstan and Western Siberia with an annual area of 17–18 Mha. This is a short-season crop grown from May to August in an extensive rainfed cropping system dominated by cereals and occasionally rotated with oilseed and legume crops. Wheat produced in the region is traded both regionally and internationally. According to FAO (https://www.fao.org/faostat/en, accessed on 1 July 2022), Kazakhstan and Russia jointly exported 42.5 Mt of wheat grain in 2020. Therefore, grain quality including health benefits or hazards is important for global food security and safety. Elemental concentration is important in defining the safety and nutrition of wheat grain. Minerals comprising wheat grain can be divided into three main groups: macroelements, calcium (Ca), potassium (K), magnesium (Mg), phosphorus (P), chlorine (Cl), and sulfur (S) which are important for starch and protein formation; toxic heavy metals, arsenic (As), cadmium (Cd), chromium (Cr) and lead (Pb), normally regulated not to exceed certain concentration; and microelements essential for plants and humans, selenium (Se), boron (B), manganese (Mn), copper (Cu), iron (Fe), molybdenum (Mo) and zinc (Zn), that can be also harmful when exceeding certain concentrations. Five microelements (Se, Mn, Cu, Fe, and Zn) along with Ca and iodine were selected as candidates for biofortification to improve the nutritional value of crops including wheat [1].

Optimization of wheat grain elemental composition attracted research and development attention from two perspectives: biofortification through genetic or agronomic enhancement of concentration of important microelements, primarily Zn, but also Fe and Se [2,3]; reduction of the concentration of toxic heavy metals such as Cd [4]. Wheat biofortification has been successfully applied for increasing Zn content in grain with commercial cultivars being grown in India and Pakistan [5,6]. A nutritional study with preschool children and women showed that consumption of high Zn biofortified wheat prevented morbidity [7]. The concept of biofortification has not been applied in wheat breeding in Kazakhstan and Russia and only a few studies have assessed the mineral composition of wheat grain produced in the region. Morgounov et al. [8] showed that the average Zn and Fe concentrations in spring wheat grain across five sites in Kazakhstan were 28 µg/g and 48 µg/g, respectively. Variation in these two elements showed a strong positive correlation. In a more recent study, Tattibayeva et al. [9] determined the concentration of toxic and essential elements in wheat grain from different regions of Kazakhstan. The maximum concentrations of As, Cd, mercury (Hg), and Pb did not exceed concentrations specified by EU, FAO, and Kazakh standards. In western Siberia, Fe, Mn, Cu, and Zn varied over ranges of 44, 38, 3, and 36 µg/g, respectively [10]. Studies on heavy metal concentrations in wheat grain in Russia were focused on individual regions like Krasnoyarsk [11], and Chelyabinsk [12] and did not identify values exceeding maximum permitted concentrations.

Three studies were undertaken recently to evaluate the variation of elemental grain composition across Northern Kazakhstan and Western Siberia and its implication for food safety, biofortification, and breeding. The ionomics analysis was conducted at the University of Nottingham in the framework of the European Plant Phenotyping Network. In the first study, 180 samples collected from wheat fields across a wide area, including eight regions of Kazakhstan and Russia were analyzed and demonstrated that toxic elements (As, Cd, Cr, Li, and Pb) were below maximum quantities as defined by FAO, EU and national regulations [13]. The presence of industry in Aktobe, East Kazakhstan, and Omsk did not have a negative effect on grain safety. The concentration of essential microelements was similar to wheat grain produced in other countries with the exception of Zn. The concentrations of this important element were around 50 µg/g in Omsk and East Kazakhstan, above the values targeted by the Harvest Plus biofortification program [2]. Even with the losses of Zn during milling, this grain could be beneficial for human health.

A genotype × environment study was based on a multilocational trial of the Kazakhstan-Siberia Network on Spring Wheat Improvement (KASIB) with 49 entries at six sites in two seasons [14]. The effect of year was least important to variation in grain ionomics composition. For several elements (P, S, Cu, Mn, and Mo), the effect of site was 2–3 times higher than the effect of genotype. The effects of genotype and site were similar for Ca, Mg, Fe, Cd, and Sr concentrations. Average broad-sense heritability was for macroelements: Mg (0.59) > Ca (0.50) > K (0.44) > P (0.30) > S (0.20); and for microelements: Zn (0.44) > Mn (0.41) > Cu (0.40) > Fe (0.38). Protein content had positive and significant genotypic correlations with Mg (0.57), P (0.60), S (0.68), Fe (0.64), Cu (0.50), Mn (0.50) and Zn (0.53). Genotypes Element-22, Lutescens-3-04-21-11, and Silach were characterized by a combination of high grain yield, relatively high protein content, and high concentration of P, S, Mn, Cu, and Zn singly or combined.

The third study focused on the spring wheat genetic diversity panel (GDP) assembled to enhance agronomic and quality traits at Omsk State Agrarian University [15]. The panel comprised three groups of germplasm: primary hexaploid synthetics (CIMMYT and Japan origin), cultivars from Kazakhstan and Russia (primarily KASIB network), and USA cultivars. The panel was genotyped using the genotyping-by-sequencing method resulting in over 46,000 SNPs. The genetic diversity study clearly separated all material into three groups: CIMMYT synthetics, Japanese synthetics, and a combined group of bread wheat germplasm from KASIB and the USA [16]. A genome-wide association study (GWAS) was conducted on yield and 26 yield-related traits, disease resistance, and grain quality traits [17]. The study identified 243 significant marker-trait associations for 35 traits that explained up to 25% of the phenotypic variance, with the most significant of these having already been used in marker-assisted breeding. Shepelev et al. [15] analyzed variation in element concentrations in the GDP. Primary synthetics had significantly higher concentrations of K and Sr, compared to the local check. The synthetics from Japan had the highest concentrations of Ca, S, Cd, and Mo. The USA cultivars had the highest concentrations of Mg and Fe. Local germplasm had average values for most elements. Superior germplasm, with high beneficial and low toxic element concentrations, was found in all groups of material.

The objective of this study was to identify the genes contributing to the variation of macro- and microelements, and trace metals in the spring wheat genetic diversity panel using GWAS and validate them using the multilocational KASIB trial mentioned above with the overall aim of developing approaches for marker-assisted breeding for grain elemental concentration.

## 2. Results

### 2.1. Agronomic Performance and Elemental Composition of Different Groups of Germplasm in GDP

**Figure 1:**
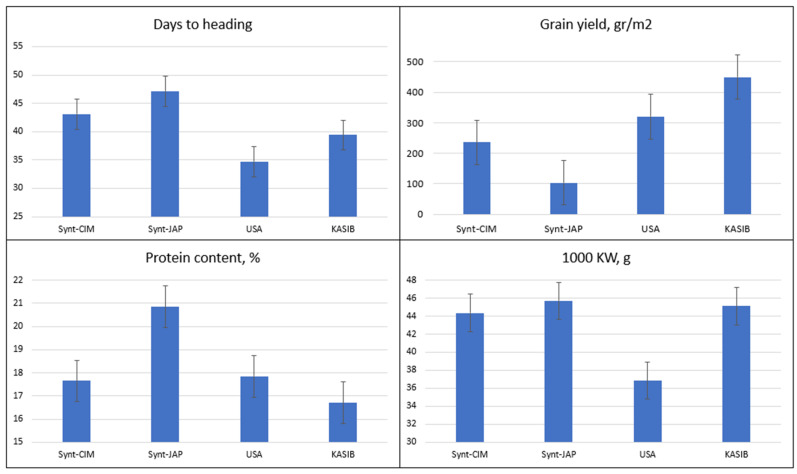
Variation for agronomic traits in different groups of genetic resources, average values for 2017–2018. Error bars indicate the standard error.

Analysis of variance (ANOVA) of the number of days to heading, TKW, grain yield, and protein content data demonstrated the high significance of genotypes, years, and their interaction except for the effect of years on TKW (Supplementary Table S1). The relative performance of different groups of genetic resources across the two years is presented in Figure 1 and for individual years in Supplementary Table S2. The number of days to heading varied from 35 (USA group) to 47 days (Japanese synthetics) while the KASIB group headed in 39.4 days. The highest grain yield was recorded for the KASIB group (449 g/m^2^) followed by USA cultivars (320 g/m^2^), CIMMYT synthetics (236 g/m^2^), and Japan synthetics (104 g/m^2^). Early maturing check Pamyati Azieva demonstrated grain yield of 399 g/m^2^ and intermediate maturing check Serebristaya 470 g/m^2^. TKW was in a range of 44.0–45.7 g for all groups though USA cultivars had smaller grain (36.9 g). The highest protein content was recorded for Japan synthetics (20%) followed by USA cultivars (17.9%), CIMMYT synthetics (16.7%), and KASIB groups (16.7%). Overall, the research panel used in the study was highly heterogeneous and contrasting especially for the vegetative period duration and grain yield.

Phosphorus had the highest concentration of all the elements in wheat grain at 5170 and 4693 µg/g in 2017 and 2018, respectively (Supplementary Table S2). The other macroelements concentrations (µg/g) in decreasing order were K (3629–3645) followed by S (2053–2063), Mg (1210–1228), and Ca (362–394). The variation between years was limited for K, S, and Mg but exceeded 10% for P and Ca. Among the microelements, Zn had the highest concentration (µg/g) in the grain (41.9–53.3 over the two years) followed by Mn (43.1–44.7), Fe (35.8–37.5), and Cu (3.73–4.67). Among three toxic trace elements, Ni had the highest concentrations at 0.212 and 0.148 µg/g in 2017 and 2018, respectively. Ni was also a highly variable element both within and between years with coefficient of variation exceeding 23.6%. Cd and Co had only low concentrations < 0.044 µg/g. Three remaining trace elements (Mo, Rb, and Sr) were also characterized by low concentrations (0.31–4.37 µg/g).

**Figure 2:**
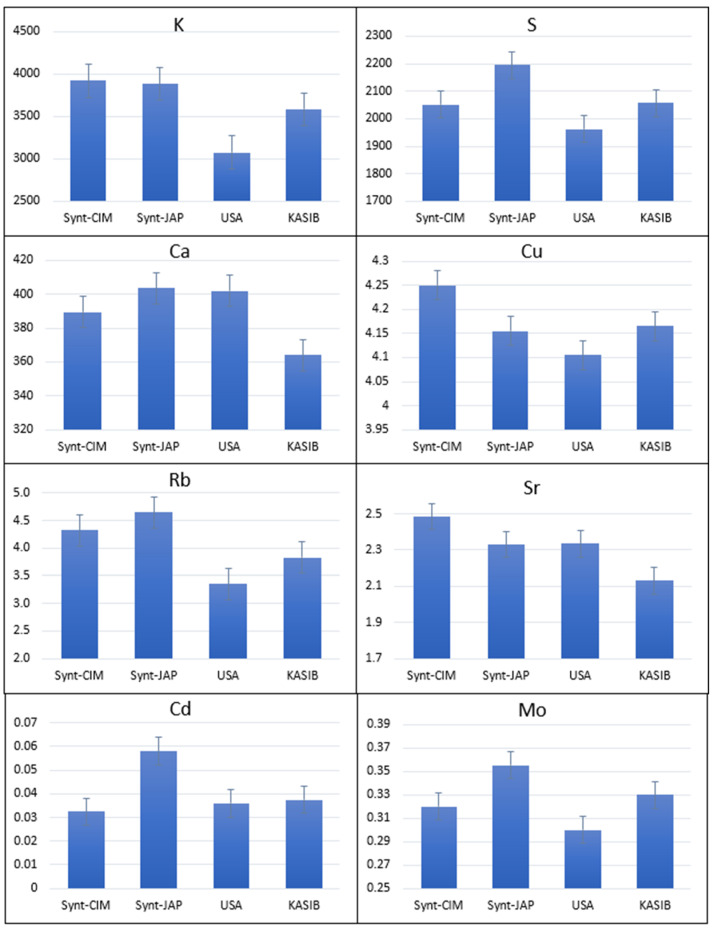
Variation for grain elemental concentration in different groups of germplasm with synthetic material demonstrating higher average values for 2017–2018. Error bars indicate the standard error.

**Figure 3:**
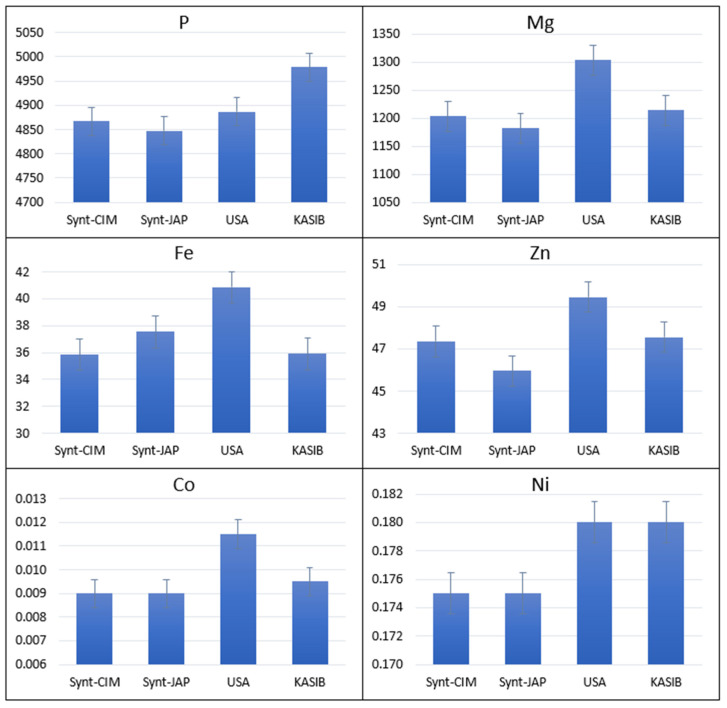
Variation for grain elemental concentration in different groups of germplasm with KASIB and USA material demonstrating higher average values for 2017–2018. Error bars indicate the standard error.

CIMMYT and Japanese synthetics had significantly higher concentrations of K (3984 and 3886 µg/g, respectively) compared to the KASIB group (3584 µg/g) (Figure 2). Japanese synthetics also had the high concentrations of Ca (403 µg/g), S (2195 µg/g), Cd (0.058 µg/g), Rb (465 µg/g) and Mo (0.355 µg/g). USA cultivars had an as high concentration of Ca as Japanese synthetics and the highest concentration of Mg (1304 µg/g) and Fe (37.6 µg/g) (Figure 3). This group was also characterized by low concentrations of K and Mo. KASIB germplasm had near average values for most elements with exception of the highest values for P (4978 µg/g) and as high Ni as USA cultivars (0.180 µg/g).

### 2.2. Results from GWAS Analysis

GWAS analysis was performed for each element separately for 2017 and 2018 data and separately for original and adjusted values. The exception was data for Ca concentration in 2017, K in 2018, and Co and Rb in both years where only original data was used since adjustment was not justified due to a lack of significant correlations between variables. In total, 55 separate GWAS analyses were conducted for element concentration. Overall, 2997 MEAs with −log_10_(*p*-value) > 3.5 and significance of effects with *p* < 0.003 were initially identified for evaluation and further selection (Table 1 and Supplementary Table S3). These 2997 MEAs belonged to 2449 SNPs and 1067 SNP regions covering all chromosomes. All MEAs were grouped into four categories: (1) SNPs with MEAs effect on the same element in the two years using either original or adjusted data (*n* = 50); (2) SNPs with MEAs effect on the same element in one year using both original and adjusted data (*n* = 197); (3) SNPs with pleiotropic effect on several elements using either original or adjusted data (*n* = 341); and (4) SNPs with effect on one element in one year using either original or adjusted data (*n* = 1861). The group 1 SNPs with effects in both years were most valuable for use as potential markers while the group 4 SNPs were least promising and excluded from further analysis.

The number of MEAs varied greatly between the elements, from 19–43 for Co, K, and Mo to 381–499 for Ni, P, S, and Mg (Table 1). Chromosome group 1 had the highest number of MEAs for Mg and P, group 3 for Ni, group 6 for Ca and S, and group 7 for Mn. The contribution of three genomes to MEAs also varied among elements. A genome-hosted highest number of MEAs for K; genome B for Mg, P, Mn, Zn, Co, Ni, and Sr; genome D for Ca and S. Group 1 SNPs with significant MEAs in the two years were identified for Ca (3), Mg (14), P (5), Cu (1), Mn (8), Cd (13), Co (1), Ni (3) and Sr (2) (Table 1 and Supplementary Table S3). There were 197 Group 2 SNPs with −log_10_(*p*-value) > 3.5 and significant effects for original and adjusted values in one year for all elements with exception of Rb. The largest number of group 2 SNPs was identified for Ni (95) followed by Ca (30) and Cd (23).

All SNPs from groups 1, 2, and 3 were screened for validation suitability using KASIB multilocational trial data. The main suitability criteria were SNP presence in 39 KASIB trial genotypes with the frequency of respective alleles at least 10%. There were 50 SNPs matching this criterion and they were subjected to validation. The remaining SNPs from groups 2 and 3 were left for future validation (Supplementary Tables S4 and S5). The SNPs from group 1 which were not subjected for validation are presented in Table 2.

For Ca SNPs on chromosomes 3B and 6D had a significant effect on concentration varying from 8.4 to 11.0% in 2017 and 7.1–9.6 in 2018. Three SNPs closely located on chromosome 1B affected Mg concentration in a range of 3.2–6.2% depending on the year. Four other SNPs on 2A, 4B, 5B, 6D, and 7B also contributed significantly to Mg concentration in wheat grain. P concentration was affected by two SNPs from the same QTL region on 6B (2.6–4.7%) and one SNP on 6D (4.0–6.2%). Only one SNP on 6D demonstrated a significant effect on Cu concentration in both years. Five SNPs (2A, 3A, 4B, and 7B) contributed to Mn concentration in a range of 4.2–8.3% in 2017 and 4.3–8.8% in 2018. Notably, the relative effects of these SNPs were comparable in both years of study. The largest SNP (2A, 2B, 3D, 4D, 5D and 7B) effects were recorded for Cd concentration: 16.8–35.3% in 2017 and 15.5–39.6%). Again, similar to the case of Mn, the effects of different SNPs were consistent over the two years, demonstrating their stability. Single chromosome 6D SNP affected Sr concentration by 16.2% in 2017 and 13.6% in 2018. Despite the fact that the effects of these SNPs were not validated, they represent good candidates for marker-assisted selection to enhance the elemental composition of wheat grain.

### 2.3. Agronomic Performance and Elemental Composition of KASIB Validation Trial

There was considerable variation in grain yield across sites and years: trials in Chelyabinsk and Novosibirsk had the highest yields exceeding 3 t/ha in both 2017 and 2018. The lowest yields were in Shortandy at around 2.5 t/ha. Four sites (Karabalyk, Omsk, Novosibirsk, and Tyumen) had high yield variability from 50% to over 100% between years. This is consistent with the large effect of weather and biotic stresses on yield stability in the region. The ANOVA indicated high significance of the three major effects: genotypes, years and locations, and all interactions (Supplementary Table S6). The protein content varied between the sites and years less than grain yield; the majority of the sites produced grain with protein content between 11 and 14%, though it exceeded 16% in Omsk in 2017 and was 10.3–10.7% in Chelyabinsk in 2017 and Tyumen in 2018. The ANOVA indicated highly significant effects for all factors and their interactions.

The average concentrations of 15 elements in the grain across years at each site are presented in Table 3. As expected, the highest concentrations (µg/g) were for macroelements, P (5037) > K (4131) > S (1616) > Mg (1218) > Ca (340). Microelements concentrations were Zn (41.4) > Mn (37.9) > Fe (34.2) and Cu (4.27). The concentrations of trace elements were Rb (3.82) > Sr (2.04) > Mo (0.377) > Ni (0.318) > Cd (0.025) > Co (0.013). For macroelements, the difference between average site minimum and maximum concentrations varied from 10.4% for K to 36% for P whereas the average difference between years across all sites varied from 6.5% for S to 10.5% for Ca. For microelements the difference between the sites varied from 28.1% for Fe to 87% for Zn; between years from 5.1% for Mn to 23.2% for Zn.

ANOVA revealed significant effects of the three main factors on all element concentrations (Supplementary Table S6). The only exception was a possible effect of year on Mg and site on Rb, which were not significant. The interaction of year and site was also highly significant for all elements suggesting that relative values and ranking of sites were different between both years. However, the interaction between the genotypes and years was not significant at *p* = 0.05 for all elements. The comparison of elemental concentration between the GWAS genetic diversity panel and KASIB multilocational validation trial demonstrated similarity between the average values but overall variation across 12 sites × years was very large, thus, allowing relevant validation of SNPs effects in different environments.

### 2.4. Validated SNPs Affecting Elemental Concentration

The list of SNPs with validated effects is presented in Table 4. Overall, 20 SNPs controlling 26 MEAs were validated for eight elements. For P there were three closely located SNPs on chromosome 1B (S1B_9711623, S1B_10111796, and S1B_13242483) with increased concentration in GDP by 2.9–3.0% and significantly increased concentration in 2–7 of the KASIB trials across sites and years by 10.6–12.0%. Two other unrelated SNPs on the same chromosome (S1B_114437220 and S1B_184771090) also significantly affected P concentration both in GDP and in KASIB across 3–4 trials. For Mo, 2A chromosome SNP (S2A_726322626) had a large effect in the GWAS panel in both years (average 12.1%) and a particularly large effect at 10 sites × years of KASIB trial (average 63.1%) For Ni there was SNP S3B_758201335, which affected the concentration in both years with a significant but small effect of 2.7% but demonstrated a significant increase in concentration by 33.3% in the three of the KASIB trials. For S there was only one SNP S4B_23355392 from group 2, which affected the concentration in 2017 in GDP (4.3%) and in six of the KASIB trials (average effect 11.6%). For Ca there were four closely related SNPs on 5A (S5A_568799967, S5A_569526776, S5A_570718644, and S5A_570788577), which affected the grain concentration of Ca by 5.0–6.0% in GWAS panel and by 9.2–13.7% at 4–6 validation trials. Another SNP (S5D_43408942) affected Ca concentration by 7.6 and 24.5%, respectively, in the two years. Strontium: two unrelated SNPs on 5A (S5A_594133493 and S5A_698528417) demonstrated significant effects in both panels and the latter affected concentration by 10.6% in GDP and by 21.1% in four of the KASIB trials.

Five pleiotropic SNPs were validated. Chromosome 1B (S1B_176291121) significantly affected both Mg and P concentrations in two panels. Two SNPs in the same QTL region of chromosome 2B affected Cd and Mn concentration (S2B_780115106), and Cd, Mn, and Zn concentrations (S2B_780665986). Their effect in KASIB trials exceeded 10.7%. Similarly, two closely located SNPs on 6B (S6B_562488824 and S6B_601138481) affected P and Zn in two panels with Zn effects exceeding 20% in two of the KASIB trials. Overall, 20 SNPs identified through GWAS analysis and validated through a multilocational trial can be recommended for use in marker-assisted selection for respective elements.

### 2.5. Annotation of SNPs Contributing to Wheat Grain Elemental Composition

The IWGSC RefSeq annotation V2.1 was used to identify the functional annotation of putative candidate genes underlying significant MEAs. The detailed description of each candidate gene underlying MEAs is listed in Table 5. A total of 47 significant MEAs were identified within putative candidate genes and of these, 87% MEAs were located in intergenic regions, 3% in the gene downstream regions, 2% in the gene upstream regions, and 1% introns (Table 5). Twenty-nine of the 47 MEAs were also enriched to other agronomic and yield-related traits. In total, 88 putative candidate genes were obtained for 47 significant MEAs (Table 5), 82 of which were tandem repeats genes. Multitrait candidate genes are the common candidate genes that influence more than one trait. Two candidate genes (TraesCS6D03G0114300 and TraesCS6D03G0114400) encoding 60S ribosomal protein L13-1 were identified for Ca and Cu elements (Table 5). Based on description information of candidate genes, many genes encoded proteins putative to the MEAs.

### 2.6. Distribution of SNP Markers among the Germplasm Groups

The SNPs with significant effects on elemental concentration in both years (Table 2) and validated through the KASIB trial (Table 4) were evaluated in regard to their frequencies in the four studied germplasm groups. (Table 6 and Supplementary Table S7). The relative share of the respective frequency of each group was taken as a basis (CIMMYT synthetics, 28.5%; synthetics from Japan, 5.1%; USA cultivars, 10.2%, and KASIB material, 56.2%) to compare the frequencies of reference SNP alleles for individual elements or their combination. The average frequencies for SNP contributing to macroelements (Ca, Mg and P) concentration was similar to the frequencies of germplasm distribution (Table 6). However, there was substantial variation among the SNPs for each element (Supplementary Table S7) which has to be taken into account while planning the crossing program. Synthetics from Japan had a low frequency of reference alleles contributing to the concentration of Cu (2.9%), Mn (1.1%), Cd (2.2%), Ni (0%), and Sr (3.3%). It was compensated by higher frequencies of these SNPs in USA cultivars (Cu) and KASIB germplasm (Mn, Cd, Ni, and Sr). For Mo, relatively higher frequencies of SNP were observed in CIMMYT synthetics and USA cultivars. The pleiotropic SNP allele contributing to Mg and P concentration had an 11.3% higher frequency in CIMMYT synthetics and 13.3% lower in KASIB material compared to germplasm distribution shares. SNP alleles affecting Cd, Mn, and Zn were more frequent in Japan synthetics and USA cultivars. Overall, the four germplasm groups possessed SNP alleles affecting all the 11 elements, the frequencies varied depending on an element and there was no dominating advantage or disadvantage of any of the material groups.

### 2.7. Superior Germplasm with Optimal Combination of Elements

The optimal grain elemental composition will combine high concentrations of beneficial macro- and microelements, and lower concentrations of trace metals including toxic elements. The genotypes used in the study were ranked for all 15 elements from highest to lowest and 20% of the top entries were marked for macro- and micronutrients and 20% of the lowest entries were marked for trace elements. The genotypes with a high concentration of macro- and microelements and low concentration of trace metals are presented in Table 7 and Supplementary Table S8. Cultivar OmGAU-100 was characterized by optimal concentration of seven elements: Ca, P, Cu, Zn, Cd, Ni, Mo, and Rb. Nine genotypes had optimal concentration of six elements: Aisberg/*Ae. squarrosa* (369)//Demir (Mg, Cu, Zn, Cd, Co., and Mo), Ukr-Od 1530.94/*Ae. squarrosa* (392) (K, P, S, Zn, Cd, Mo, and Sr), Langdon/KU-2075 (P, Cu, Fe, Mn, Cd, and Rb), Langdon/KU-2093 (K, Mg, S, Zn, Co, and Sr), Freyr (P, Zn, Cd, Co, Ni, and Mo), Lutescens-48-204-03 (P, Zn, Cd, Co, Ni, and Mo), Lutescens-1103 (Ca, Mg, Mn, Ni, and Mo), Novosibirskaya-41 (P, Cu, Cd, Co, Ni, and Mo) and Silach (Ca, Mg, Fe, Ni, and Rb). The genotypes with an optimal concentration of elements were identified across all four groups of germplasm including synthetic wheat, KASIB, and USA cultivars.

Selected synthetic wheats and USA cultivars were characterized by lower grain yield of 11.5–73.8% compared to local check and higher protein content by up to 30.5% with variable TKW (Table 7). The high-yielding KASIB entries combined high protein content, large grain, and optimal concentration of several elements: Element-22 (Ca, P, S, Cu, and Ni), Lutescens-6-04-4 (K, S, Cu, Mn, and Cd), Uralosibirskaya (K, Mg, S, Fe) and Silach (Ca, Mg, Fe, Ni, and Rb). The superior germplasm identified during this study offers a choice of parents for a targeted crossing program along with the information on SNPs contributing to the concentration of important elements.

## 3. Discussion

### 3.1. Elements and Underlying Physiology and Biochemistry

The ionome is defined as the mineral nutrient and trace element composition of an organism and represents the inorganic component of cellular and organismal systems [32]. Ionomics, the study of the ionome, involves the quantitative and simultaneous measurement of the elemental composition of living organisms and changes in this composition in response to physiological stimuli, developmental state, and genetic modifications. The advantage of the ionomics approach is that complex biochemical processes in plants are reflected through simple concentrations of macro-, micro-, and trace elements. This makes the evaluation of wheat grain or other products relatively simple when the enhancement target is a single element. Similar to the case of biofortification targeting increase of Ca, Fe, Cu, Mn, or Zn concentration, ionomics provides an integrated approach and a value of the element which can be used for the selection of genotypes or technological practices independently of the underlying physiological and biochemical processes. However, this is also one of the limitations of this approach as it is difficult to single out specific compound/s or biochemical cycle/s affecting the element concentration. For this reason, the present study does not attempt the discussion of the underlying biochemistry and physiology though recognizing its importance.

### 3.2. Differences between the Elements on Number of SNPs

The key advantage of the present study was a wide range of 15 elements included in the analysis of genetic control of their concentration in grain. The number of significant MEAs and SNPs affecting a particular element is important for designing and applying marker-assisted selection. There was great variation in this respect with the number of MEAs varying from 20–40 for K, Co, and Mo to over 380 for Mg, P, S, and Ni. However, it is important to determine the possible factors making some elements more suitable for selection using genomic tools while others are less suitable. An assumption can be made that macroelements primarily involved in structural grain composition including protein would have less number of MEAs with possibly stronger effects as compared to microelements involved in a number of regulatory fermentative compounds or trace metals that are involved in specific cell processes. However, the reality is that Ni was identified as an element with a number of MEAs and reliable SNPs which can be used for selection. Taking into consideration SNPs with stable significant MEAs expression in both years as well as the SNPs with validated effect, for five elements (K, Fe, Co, Ni, Rb) there were no SNPs identified with significant reliable effects. The ranking of the remaining elements using the number of reliable MEAs was P (11 MEAs listed in Table 2 and Table 4), Mg (9), Cd (9), Ca (7), Mn (7), Zn (3), Sr (3), S, Cu, Ni and Mo (1 each for the latter 4 elements). Previous ionomics study of KASIB multilocational trial [14] rated the elements according to broad-sense heritability values: for macroelements, Mg (0.59) > Ca (0.50) > K (0.44) > P (0.30) > S (0.20); for microelements, Zn (0.44) > Mn (0.41) > Cu (0.40) > Fe (0.38); and for trace elements, Mo (0.56) > Sr (0.55) > Co (0.49) > Cd (0.44) > Ni (0.37) > Rb (0.31). Obviously, there is some confirmation of the elements’ suitability for genetic enhancement based on higher H^2^ values and the number of MEAs with significant effects identified in the present study; elements Mg, Ca, Cd and Mn.

### 3.3. Relation to SNPs Identified in Other Publications

The mineral content of wheat grain attracts growing scientific attention due to the focus on improving nutritional value. Wheat biofortification by Iron and Zinc has been recently reviewed by Wani et al. [33], and Kamaral et al. [34] summarizing the results and perspectives for the future. There are numerous recent GWAS studies on macro-, micro-, and trace elements by Rathan et al. [35], Wang et al. [29], Hao et al. [36], Alomari et al. [37], and El-Soda and Aljabri [38]. The present study contributes knowledge on the genetic control of 11 elements using a diverse set of germplasm phenotyped in Siberia. The important question is if the MEAs and SNP identified in the study have been reported before in connection with mineral content or other traits. The majority of the loci identified in the present study are new and unique in their effect on wheat elemental composition. This originates from the uniqueness of the germplasm which represents KASIB short-season high latitude wheat which has not previously been widely studied. The rainfed environment of Western Siberia is also unique and different from similar spring wheat areas in North America or Scandinavia [39]. However, several SNPs identified in the present study have been reported previously.

The SNPs on 5A (S5A_569526776) affecting Ca concentration were reported to contribute to herbicide metribuzin tolerance identified in Gulf Atlantic Wheat Nursery in the USA [27]. Alomari et al. [40] conducted a GWAS analysis of Ca concentration in European wheats and also identified the most significant SNP (RAC875_c8642_231) on chromosome 5A (114.5 cM). The gene underlying this marker encodes a cation/sugar symporter. The second significant Ca concentration locus (wsnp_Ex_c20899_30011827) on the same chromosome (117.7 cM) carries a gene that encodes an AP2-type transcription factor. The relationship between these two loci and the SNPs on 5A identified in the present study needs to be investigated.

The present study identified SNP S2A_738732586 contributing to Magnesium concentration. The same SNP was identified in CIMMYT synthetics collection contributing to Cu concentration based on both years of study in Turkey [31]. Chromosome 4B SNP S4B_64816370 affecting Mg concentration also had a pleiotropic effect on grain circularity and length at the GWAS analysis of the same material and in the same years for agronomic traits [25]. SNP S6B_610963076 contributed to P concentration in the present study and also was identified as having an effect on stem diameter in the study in Turkey [31]. The related gene TraesCS6B01G346900-TraesCS6B01G347000 controls disease resistance protein and F-box protein-like. The SNP S5A_698528417 contributed to Strontium concentration in the present study and also was identified as QTL for area per spike in the USA winter wheat study [28]. Importantly, none of the SNPs listed above had an effect on the same elements in other studies but rather demonstrated pleiotropic nature. This once again underlines the fact that the connection between the element concentration in grain and its underlying physiological and biochemical processes is not straightforward and not easy to trace.

### 3.4. Breeding Approaches Based on the Study Results

The spring wheat breeding framework in northern Kazakhstan and western Siberia is largely based on traditional approaches with limited application of modern tools. The majority of cultivars grown in the region represent tall, day-length sensitive types with limited genetic diversity [41]. The concept of biofortification through genetic improvement has not yet been incorporated into practical breeding efforts. The series of ionomics studies with spring wheat in the region allows the development of a breeding strategy and methodology to enhance the elemental composition. Two previous ionomics studies in Northern Kazakhstan and Western Siberia [14,15] demonstrated a large effect of protein content on the concentration of both macro- and microelements. Concentrations of Fe, Mn, and S were positively correlated with protein content on both environment and genotype levels. For Mg, P, Cu, and Co, environmental correlations with protein content were low and not significant whereas genotypic correlations were positive and relatively high (0.50–0.62). However, this GWAS study did not identify any pleiotropic effects of protein content on any of the elements. This could be due to the adjustment of element concentration values using multiple regression analysis. Nevertheless, the selection of genotypes with high protein content would be the logical choice to enhance grain nutritional value.

The analysis of genotype by environment interactions for the KASIB trials showed that genetic improvement for all elements would be highly affected by the uniformity of experimental fields [14]. Broad sense heritability values demonstrated substantial variation between sites and years for all elements. The highest H^2^ across both years was observed in Tyumen. If breeding efforts targeting elemental composition are to be undertaken, regional cooperation would be essential to design a selection program with an emphasis on elements with high genetic variation to be evaluated at sites with uniform fields. The availability of high throughput and high precision analytical facilities in the region would be an essential component of such a program.

Primary synthetic wheat developed from crosses of durum wheat with *Ae. tauschii* has been reported as a source of high concentration of microelements including Fe and Zn [3,42]. In the present study, based on the original values, primary synthetics from Japan also demonstrated a high concentration of a number of elements including Ca, Mg, P, S, Fe, and Zn. However, after adjustment using multiple regression, the synthetic wheat germplasm largely lost its advantage. Superior germplasm combining high concentration of macro- and microelements and low concentration of trace elements was identified in all germplasm groups including KASIB material, USA cultivars, and both synthetics groups. The genotypes with a favorable concentration of five and six elements were also identified in all germplasm groups. There is sufficient diversity for elemental composition and agronomic traits among selected genotypes to plan targeted crossing and selection programs.

The crossing strategy to incorporate and combine optimal concentration of a wide range of elements depends on the nature of the germplasm. Synthetic wheat with low yield and a number of undesirable traits like spike threshability requires a top and back crossing scheme to transfer useful traits while maintaining and improving grain yield. Several synthetics from the present study possess resistance to leaf, stem rust, and powdery mildew [43] making them attractive as parental material. Pathogen resistance, short stature, and earliness are additional positive traits of USA cultivars for improvement of Siberian wheat for ionome profile. Simple crosses and the development of a large population may be sufficient to combine positive traits of the KASIB and USA material. However, the back- and top-crosses with local material may also be efficiently used. A crossing program within the KASIB breeding network would be straightforward based on simple crosses and consequent selection.

The molecular markers identified in the present study for 11 elements may require additional validation within the breeding process considering that the crosses made back in 2017 and 2018 are now in preliminary yield tests and can be traced back to specific parents and SNPs. They provide an essential platform for fast and efficient selection to increase the concentration of beneficial elements and decrease the harmful minerals, thus, contributing to food security and safety of the locally grown wheat grain.

## 4. Materials and Methods

### 4.1. GWAS Genetic Diversity Panel Material, Field Experiments

The GDP panel comprised 135 entries including two checks as listed in Supplementary Table S9. The research material included 37 primary hexaploidy synthetics from CIMMYT originated from crosses between Ukrainian winter durum wheat cultivars and several accessions of *Aegilops tauschii* from the CIMMYT genebank. The development of the synthetics through targeted selection under abiotic and biotic stresses was described by Morgounov et al. [44]. Eight primary synthetics developed by Kyoto University in Japan [45] comprised second group. The USA cultivars (14 in total) included hard red spring wheat entries primarily from University of Minnesota and Syngenta. Material from KASIB was represented by new cultivars and breeding lines (74 in total). The two checks were widely grown spring wheat cultivars in the Omsk region, cultivars Pamyati Azieva and Serebristaya, representing early and intermediate maturity groups, respectively. The main contributors of KASIB germplasm were Omsk State Agrarian University with 17 entries and Omsk Agrarian Research Center with 14 entries. The KASIB group also included 17 cultivars and breeding lines from Kazakhstan.

The trial was planted in the experimental field of Omsk State Agrarian University (55.04° N, 73.36° E) as a randomized complete block design with plots of 1 m^2^ and four replicates in 2017 and 2018. Soil of the experimental field was meadow chernozem with 5% organic matter content and average availability of NPK. Preceding crop was black fallow. Spring soil preparation comprised harrowing in early May followed by shallow cultivation and harrowing in mid-May. Planting took place between 15–20 May in both years. The trials were harvested in the first week of September. No fertilizer or fungicides were applied. Weeds were controlled by application of a common herbicide after tillering stage in mid-June. The field observations included agronomic traits including heading dates, disease evaluations, yield, and yield components. CIMMYT Wheat Physiology Manual [46] was used as a guide for germplasm evaluations for all traits and diseases. The protein content in the grain was determined using Infratec FOSS 1841.

Weather conditions in 2017 were characterized by 1 °C higher temperature in May-August compared to long-term observations and lower rainfall resulting in drought and yield reduction (Supplementary Table S10). In 2018 the temperature was cooler by 1.2 °C and rainfall 24 mm higher. A high level of stem and leaf rust was observed on susceptible entries.

### 4.2. Validation Panel KASIB Trial Material, Field Experiments

KASIB trial comprised 39 entries including three checks. All of them were included in GWAS GDP and marked in Supplementary Table S9. The research material included 13 genotypes from seven breeding programs in Kazakhstan and 26 genotypes from 10 Russian institutions. Most of the material included in KASIB trial were advanced breeding lines and several new cultivars. The trial included three checks of widely grown spring wheat varieties (Element-22, Pamyati Azieva, and Omskaya-35) with variable maturity ranges. The trial was planted at six sites in Kazakhstan and Russia in randomized complete block designs with plots of 3–5 m^2^ and two or three replicates. The seeds used for the first year of the trial originated in the institutions which submitted the germplasm. In the second year, all KASIB cooperators used their own seed. The field observations included three adaptation traits (heading and maturity dates, plant height) and disease evaluations under natural infection. Grain yield was recorded after combine harvesting. CIMMYT Wheat Physiology Manual [46] was used as a guide for germplasm evaluations for all traits and diseases.

Six KASIB testing sites were located between the southernmost Shortandy at 51.6° N and the northernmost Tyumen at 57.1° N or around 800 km. The westernmost site Chelyabinsk (60.7° E) was 1400 km from the easternmost site Novosibirsk (83.6° E). The large distances between the sites are reflected in soil, climate, and weather variation (Supplementary Table S10). There is a clear tendency for cooler summer temperatures and higher rainfall when moving from south to north: the lowest temperature and highest rainfall was recorded in both years in Tyumen followed by Novosibirsk and Chelyabinsk. In 2017 drought occurred in Shortandy and Omsk with rainfall below 70% of the long-term average whereas Novosibirsk had 23% higher than average rainfall. In 2018 average rainfall in May-August at all six sites exceeded the long-term average by 22.1%. Overall weather variation at 12 locations × years allowed a detailed characterization of the germplasm.

The soils across this large region were represented by various types of chernozem: ordinary (Karabalyk), leached (Shortandy, Novosibirsk, Tyumen), carbonate (Chelyabinsk) and meadow (Omsk). The soil was generally deep (60–80 cm) and fertile with humus content exceeding 4.5–5.0%. Chelyabinsk soils were characterized by slight salinity. The nitrate nitrogen availability was low in Shortandy, Chelyabinsk, and Omsk. Phosphorus concentration was intermediate in Karabalyk and high at other sites. Potassium availability was low in Novosibirsk, intermediate in Chelyabinsk, and high at other sites.

The trials were conducted at experimental fields in Kazakhstan at the Karabalyk Experimental Agricultural Station (Karabalyk, Kostanay Region) and Scientific-Production Center of Grain Farming named after A.I. Barayev (Shortandy, Akmola Region), and in Russia at the Chelyabinsk Research Institute of Agriculture, (Chebarkul, Chelyabinsk Region), Omsk State Agrarian University (Omsk), Siberian Research Institute of Plant Production and Breeding (Novosibirsk) and Northern Trans-Ural State Agricultural University, Tyumen. KASIB trial was planted along with the breeding material and followed common agronomy practices for spring wheat at each location. Prior to each trial, the field was under bare (black) fallow. Spring soil preparation was done by cultivation and harrowing. Planting was conducted at optimal dates in mid-May. The seeding rate was 400–450 seeds/m^2^. Weeds were controlled using common herbicides. The agronomy practices applied in the breeding fields to large extent followed the common local commercial practices. Normally fertilizers are not applied on wheat field following bare fallow and this was the case for KASIB trial.

### 4.3. Grain Ionomics Analysis, Values Adjustment and Analysis of Variance

The ionomics phenotyping platform at the University of Nottingham (UK) conducted the ionomics analysis for both GWAS and validation panels. Kazakh Research Institute of Farming and Crop Production (Almaty, Kazakhstan) received and processed all the grain samples, cleaned, and analyzed for protein content using Infratec FOSS 1841 at 14% moisture. Subsamples were sent to the Ionomics Facility at the University of Nottingham and the ionomics analysis was performed using state of the art Perkin Elmer NexION 2000 inductively coupled plasma mass spectrometer (ICP-MS). The samples were prepared for the ICP-MS analysis in the adjoining high throughput preparation laboratory. Wheat grains were transferred into the Pyrex test tubes, weighted, and initially predigested with 1 mL concentrated trace metal grade nitric acid Primar Plus (Fisher Chemicals, Zurich, Switzerland) spiked with 20 μg/L of indium internal standard, for approximately 20 h at room temperature. Indium was added to the nitric acid as an internal standard for assessing errors in dilution, variations in sample introduction, and plasma stability in the ICP-MS instrument. After the pre-digestion step, samples were transferred into DigiPREP MS dry block heaters (SCP Science, Baie D’Urfé, Québec; QMX Laboratories, Thaxted, UK) and digested for 4 h at 115 °C. After cooling down, 1 mL of trace metal grade hydrogen peroxide (Primar, Fisher Chemicals) was added to the tubes, and samples were digested in dry block heaters for additional 2 h at 115 °C and then diluted to 10 mL with 18.2 MΩcm Milli-Q Direct water (Merck Millipore, Burlington, Massachusetts, USA). Five replicate analyses were conducted for each sample from each replication and the mean value represented the sample final readings.

The ionomics results were obtained for 23 elements: macroelements, Ca, K, Mg, P, and S; microelements, B, Fe, Cu, Na, Mn, and Zn; toxic trace elements, As, Cd, Co, Cr, Ni, Pb, and Se; and trace elements, Li, Mo, Rb, and Sr. The concentrations were either insignificant or below the limit of quantification for B, Na, As, Cr, Pb, Se, Li, and Ti, therefore, these elements were excluded from the analysis. For the remaining 15 elements, the concentrations were normalized to the weight of the samples and expressed in µg/g of dry weight.

Three groups of GWAS GDP varied substantially in grain yield affecting the protein content and elemental concentration. Correlations between individual element concentrations and the other variables, viz., grain yield, 1000 kernel weight (TKW), protein content, and macroelements concentrations, were calculated using Microsoft Excel. The correlation analysis results (Supplementary Table S11) were used to adjust the original element concentration values using multiple regression on the following traits: grain yield, protein content, TKW, and concentrations of Ca, K. Mg, P, and S. The concentration of each element was adjusted only for the traits with significant correlation. Some elements (Co and Rb) did not correlate with any variable and, therefore, no adjustments were made. Some elements (Ca, K and Fe) did not correlate with other traits in one year but correlated in another year, and adjustments were made only for the year with significant correlations. For all other elements, adjustments were made in both years and the number of variables in regression varied from one to five. Both original and adjusted values were used for GWAS analysis. No adjustments were made for the KASIB trial due to comparable grain yield and protein content.

Factorial ANOVA (genotype × sites for GWAS panel and genotype × sites × year for KASIB trial) was used for statistical analysis for all agronomic traits and for each element independently using R software package version 3.4 [47]. For the GWAS panel, all analyses were conducted separately for the original and adjusted values. Broad-sense heritability (H^2^) was estimated for each element in individual KASIB trials (for each year separately) based on the ANOVA results.

### 4.4. DNA Analysis, GWAS and Validation Methodology, Genes Annotation

Genomic DNA was extracted from fresh young leaves (approx. 14 days after sowing) using BioSprint^®^ 96 Plant Kit (QIAGEN). The GBS libraries were constructed in 96-plex following digestion with the restriction enzymes PstI and MspI at Wheat Genetics Resource Center at Kansas State University (Manhattan, KS, USA). SNP calling was performed using TASSEL v.5.2.40 GBS v2 Pipeline [48] with a physical alignment to the Chinese spring genome sequence (RefSeq v1.0) [49]. The identified SNPs were filtered for minor allele frequency (MAF) less than 5% and missing rate of more than 20%.

Genome-wide association analyses based on the high-quality SNPs were conducted using the FarmCPU model implemented in R package “rMVP” (https://github.com/xiaolei-lab/rMVP, accessed on 21 June 2022). The top two principal components (PC1 and PC2) and kinship were chosen as covariates to control Type-I error. The principal components were internally calculated, then the package function *mvp.data.kin* was used to estimate kinship matrix. The multiple tests based on Bonferroni correction are often too conservative, so many MEAs may not pass the stringent criterion of the significant test. To balance the Type-I and Type-II errors, a −log_10_(*p*) of 3.5 was adopted as the threshold for significant MEAs.

Significant SNP markers on elements concentration in two years were screened for validation suitability following one criterion, presence in KASIB trial with frequency of reference or alternative allele exceeding 10%. Overall, 50 SNPs were selected for validation following this criterion. The significance of difference between average values of two groups of germplasm with reference and alternative alleles was estimated for each of 12 site × year combinations (6 sites in 2017 and 2018) using Welch’s *t*-test in R software [47].

To further understand the genetic control of elemental composition, position-dependent gene search strategy was processed to link significant MEAs with putative candidate genes. Functional annotation and the effect of significant MEAs were performed using SnpEff version 4.3t (http://snpeff.sourceforge.net accessed on 15 June 2022). Functional enrichment analysis of candidate genes harboring significant MEAs was conducted at http://wheat.cau.edu.cn/TGT/index.html (accessed 30 June 2022). Gene descriptions as indicated by the IWGSC RefSeq V2.1 database were used in this study.

## Figures and Tables

**Table 1:** Results of GWAS analysis and identification of significant MEA for grain concentration of 15 elements in 2017 and 2018 in diversity panel.

	Elements															
	Ca	K	Mg	P	S	Cu	Fe	Mn	Zn	Cd	Co	Ni	Mo	Rb	Sr	All
	Number of significant marker-element associations (Groups 1–4)															
All	229	32	499	394	399	111	63	224	212	243	19	381	43	78	212	2997
Chr. 1	35	2	180	165	13	2	18	18	40	33	0	30	6	3	40	548
Chr. 2	5	5	49	42	47	17	14	20	46	63	1	45	11	8	46	384
Chr. 3	9	6	41	38	28	26	6	23	7	31	5	111	9	31	7	372
Chr. 4	8	2	31	21	58	19	5	67	33	35	0	23	3	2	33	307
Chr. 5	51	3	110	41	50	8	7	20	30	17	3	59	4	16	30	436
Chr. 6	108	7	24	54	189	23	6	12	47	23	9	62	3	8	47	598
Chr. 7	13	7	64	43	14	16	7	64	9	41	1	51	7	10	9	352
Gen. A	58	17	203	99	130	35	25	66	47	88	3	94	20	31	47	937
Gen. B	33	10	222	178	119	40	16	140	97	93	15	154	10	26	97	1191
Gen. D	138	5	74	117	150	36	22	18	68	62	1	133	13	21	68	869
	Number of SNPs with significant effects															
Group 1	3	0	14	5	0	1	0	8	0	13	1	3	0	0	2	50
Group 2	30	2	2	4	11	2	2	5	15	23	0	95	4	0	2	197

**Table 2:** SNPs with significant effects on single elements in two years.

#	SNP	QTL Region	Element	REF	ALT	2017			2018		
						Effect, µg/g	Effect, %	−log_10_(*p*-Value) > 3.5	Effect, µg/g^−1^	Effect, %	−log_10_(*p*-Value) > 3.5
1	S3B_807804964	442	Ca	A	G	39.9	11.0	3.57	37.7	9.6	3.67
2	S6D_27846508	878	Ca	T	A	30.4	8.4	4.05	28.0	7.1	3.75
3	S1B_468389275	112	Mg	C	A	38.2	3.2	3.78	60.7	4.9	3.71
4	S1B_470419196	113	Mg	C	T	38.2	3.2	3.99	60.0	4.9	3.85
5	S1B_483598145	114	Mg	T	G	51.4	4.2	4.24	75.6	6.2	3.63
6	S2A_738732586	227	Mg	T	G	48.2	4.0	3.81	80.8	6.6	4.15
7	S4B_64816370	562	Mg	C	T	46.7	3.9	3.77	75.4	6.1	3.83
8	S5B_679675578	738	Mg	T	C	33.3	2.7	3.58	21.2	1.7	4.38
9	S6D_469161928	958	Mg	C	G	32.9	2.7	4.03	17.5	1.4	3.78
10	S7B_723334278	1040	Mg	C	T	−52.7	−4.4	3.78	−80.8	−6.6	3.50
11	S6B_610963068	852	P	G	A	−246	−4.7	3.80	−131	−2.8	3.67
12	S6B_610963076	852	P	G	T	−135	−2.6	3.88	−131	−2.8	3.67
13	S6D_376894590	932	P	G	A	−319	−6.2	3.87	−188	−4.0	4.49
14	S6D_29369738	879	Cu	C	G	0.320	6.9	3.59	0.281	7.5	3.78
15	S2A_24200649	206	Mn	G	A	−3.22	−7.5	4.25	−3.13	−7.0	3.96
16	S3A_697506434	374	Mn	G	T	−2.41	−5.6	3.88	−2.35	−5.3	3.66
17	S4B_603519569	579	Mn	C	A	−3.52	−8.2	3.56	−3.53	−7.9	3.98
18	S7B_574853540	1015	Mn	G	T	−1.82	−4.2	3.60	−1.93	−4.3	3.94
19	S7B_720831474	1039	Mn	C	T	−3.60	−8.3	4.15	−3.93	−8.8	4.83
20	S2A_751844369	231	Cd	A	G	0.008	18.9	3.87	0.007	21.0	4.88
21	S2B_772063522	297	Cd	C	G	0.016	35.3	4.61	0.010	31.5	3.87
22	S2B_88259062	246	Cd	G	T	0.007	16.8	3.95	0.005	15.5	3.53
23	S3D_550209436	482	Cd	G	A	−0.008	−18.1	4.03	0.012	−39.6	3.72
24	S4D_11471805	592	Cd	C	T	0.010	23.0	3.87	0.007	24.0	4.25
25	S5D_486749507	768	Cd	C	T	0.014	32.8	4.50	0.009	28.3	3.55
26	S7B_677743542	1033	Cd	A	G	0.008	19.0	3.69	0.006	19.1	3.85
27	S6D_454012454	953	Sr	G	A	0.338	16.2	3.62	0.329	13.6	3.79

**Table 3:** The grain elements concentration of KASIB trials across sites and years in 2017–2018.

Element	Mean Concentration Across 2017–2018, µg/g						LSD 0.05
	Karabalyk, KZ	Shortandy, KZ	Chelyabinsk, RU	Omsk, RU	Novosibirsk, RU	Tyumen, RU	
Ca	306	319	309	373	391	341	29
K	4176	3908	4276	4022	4093	4313	185
Mg	1314	1089	1225	1293	1213	1177	59
P	4944	3960	5343	5387	5269	5320	360
S	1818	1948	1719	1949	1829	1515	111
Cu	3.82	5.28	3.46	4.67	4.19	4.20	2.6
Fe	34.6	32.5	33.7	39.7	33.8	31.0	0.45
Mn	40.8	42.0	34.1	41.8	38.6	30.3	3.1
Zn	37.2	26.9	44.3	50.3	44.9	44.9	6.2
Cd	0.021	0.025	0.026	0.033	0.030	0.018	0.004
Co	0.020	0.027	0.008	0.011	0.007	0.004	0.006
Ni	0.402	0.249	0.541	0.257	0.123	0.336	0.091
Mo	0.461	0.571	0.190	0.290	0.268	0.488	0.10
Rb	1.88	1.76	4.07	3.28	7.06	4.88	1.22
Sr	2.83	2.34	1.67	2.05	1.69	1.65	0.34

**Table 4:** SNPs with significant effects on elemental composition validated using KASIB trials.

#	SNP	Element	QTL Region	Ref. SNP	Alt. SNP	GWAS Panel *				KASIB Validation Trial **		
						Year	Effect, µg/g	%	−log_10_(*p*-Value) > 3.5	No. of Sites	Effect, µg/g	%
1	S1B_9711623	P	58	C	T	2017	157	3.0	4.95	2	598	12.0
2	S1B_10111796	P	58	G	T	17	149	2.9	4.69	7	514	10.0
3	S1B_13242483	P	59	A	G	17	150	2.9	4.89	4	539	10.6
4	S1B_114437220	P	82	T	C	17	156	3.0	4.87	3	385	7.3
5	S1B_176291121	Mg	96	A	G	17	−31.8	−2.6	4.01	1	−75	−5.6
		P	96	A	G	17	152	2.9	4.79	3	608	12.1
6	S1B_184771090	P	99	T	C	17	153	2.9	4.76	4	509	9.9
7	S2A_726322626	Mo	223	A	G	17–18	0.041	12.1	9.29	10	0.25	63.1
8	S2B_780115106	Cd	300	A	G	18	−0.003	−9.7	4.24	4	−0.09	−26.6
		Mn	300	A	G	17	−1.96	−4.5	5.19	6	−4.51	−10.7
9	S2B_780665986	Cd	300	T	C	17–18	0.001	3.2	4.50	4	0.09	28.1
		Mn	300	T	C	17	−2.06	−4.8	5.57	4	−4.68	−11.5
		Zn	300	T	C	17	−3.01	−5.6	3.90	1	−7.0	−13.5
10	S3B_758201335	Ni	432	A	G	17–18	0.004	2.7	4.11	3	0.054	33.4
11	S4B_23355392	S	561	C	T	17	87.9	4.3	5.25	6	203	11.6
12	S5A_568799967	Ca	658	C	G	18	−23.2	−5.9	6.28	6	−44	−13.7
13	S5A_569526776	Ca	658	C	T	18	19.8	5.0	4.78	4	38	12.2
14	S5A_570718644	Ca	659	A	G	18	−21.1	−5.3	4.81	5	−28	−9.2
15	S5A_570788577	Ca	659	T	G	18	−23.9	−6.0	4.97	5	−40	−13.1
16	S5A_594133493	Sr	661	A	T	18	−0.158	−6.5	4.86	5	−0.225	−11.2
17	S5A_698528417	Sr	677	C	G	17–18	0.256	10.6	5.69	4	0.475	21.1
18	S5D_43408942	Ca	749	A	G	17–18	30.1	7.6	3.61	3	88	24.5
19	S6B_562488824	P	846	C	T	17	−308	−5.9	3.98	2	−552	−10.7
		Zn	846	C	T	17	−4.66	−8.7	3.56	2	−10.1	−20.6
20	S6B_601138481	P	848	C	T	17	−308	−6.0	3.98	1	−709	−14.4
		Zn	848	C	T	17	−4.66	−8.7	3.56	2	−10.1	−22.4

* Average effect for the specified year or both years. ** Number of sites × years (out of 12) where the SNP effect was significant with *p* < 0.05; average effect for the specified number of sites × years with significant effects.

**Table 5:** Annotation of SNPs with effects on wheat grain elemental composition.

SNP	Position (Mb)	Annotation	Gene ID *	Description	References
S1B_9711623	9.711623	Intergenic region	TraesCS1B03G0039600-TraesCS1B03G0040000	G-type lectin S-receptor-like serine/threonine-protein kinase At2g19130, Putative 12-oxophytodienoate reductase 4	[18,19]
S1B_10111796	10.111796	Upstream gene variant	TraesCS1B03G0041500	Rust resistance kinase Lr10	
S1B_13242483	13.242483	Intergenic region	TraesCS1B03G0051800-TraesCS1B03G0051900	Bowman-Birk type trypsin inhibitor	
S1B_114437220	114.43722	Intergenic region	TraesCS1B03G0269300-TraesCS1B03G0269500	Chitinase 10, Hydrophobic protein LTI6B	
S1B_176291121	176.291121	Intergenic region	TraesCS1B03G0363900-TraesCS1B03G0364700	LRR receptor-like serine/threonine-protein kinase	
S1B_184771090	184.77109	Intergenic region	TraesCS1B03G0374100-TraesCS1B03G0374900	Pre-mRNA splicing factor SR-like 1, E3 ubiquitin-protein ligase	
S1B_468389275	468.389275	Intergenic region	TraesCS1B03G0736700-TraesCS1B03G0737500	NADH dehydrogenase [ubiquinone] iron-sulfur protein 2	
S1B_470419196	470.419196	Intergenic region	TraesCS1B03G0739000-TraesCS1B03G0739200	Uncharacterized protein, Autophagy-related protein 18 h	
S1B_483598145	483.598145	Intron variant	TraesCS1B03G0758900	Guanosine nucleotide diphosphate dissociation inhibitor 1	[20]
S2A_24200649	24.200649	Intergenic region	TraesCS2A03G0098800-TraesCS2A03G0099400	Bidirectional sugar transporter SWEET6b	[19]
S2B_88259062	88.259062	Intergenic region	TraesCS2B03G0283000-TraesCS2B03G0283400	Probable pectinesterase 53	[19]
S2B_772063522	772.063522	Intergenic region	TraesCS2B03G1432900-TraesCS2B03G1433300	BTB/POZ and MATH domain-containing protein 2	[21]
S2B_780115106	780.115106	Downstream gene variant	TraesCS2B03G1459800	N/A	[21]
S2B_780665986	780.665986	Intergenic region	TraesCS2B03G1462400-TraesCS2B03G1462900	N/A	[21]
S7B_574853540	574.85354	Intergenic region	TraesCS7B03G0857100-TraesCS7B03G0858100	Eukaryotic initiation factor 4A, Cadmium/zinc-transporting ATPase HMA2	
S7B_677743542	677.743542	Intergenic region	TraesCS7B03G1084500-TraesCS7B03G1085100	MMS19 nucleotide excision repair protein homolog, Ethylene-responsive transcription factor 12	[19,22]
S7B_720831474	720.831474	Downstream gene variant	TraesCS7B03G1209100	Receptor-like cytoplasmic kinase 176	[18,19]
S3A_697506434	697.506434	Intergenic region	TraesCS3A03G1072400-TraesCS3A03G1072800	N/A	
S3B_758201335	758.201335	Intergenic region	TraesCS3B03G1233100-TraesCS3B03G1234800	Amino acid transporter AVT1I, Glutathione transferase GST 23	[19,20,22,23]
S3B_807804964	807.804964	Intergenic region	TraesCS3B03G1388900-TraesCS3B03G1389300	Probable disease resistance protein	[21]
S3D_550209436	550.209436	Intergenic region	TraesCS3D03G0963300-TraesCS3D03G0963700	LRR receptor-like serine/threonine-protein kinase EFR, Serine carboxypeptidase 1	
S4B_23355392	23.355392	Intergenic region	TraesCS4B03G0059500-TraesCS4B03G0060200	E3 ubiquitin-protein ligase UPL7	[22,24]
S4B_64816370	64.81637	Intergenic region	TraesCS4B03G0150900-TraesCS4B03G0151600	N/A	[25]
S4B_603519569	603.519569	Intergenic region	TraesCS4B03G0818900-TraesCS4B03G0819000	Mitogen-activated protein kinase kinase 1	
S4D_11471805	11.471805	Intergenic region	TraesCS4D03G0040900-TraesCS4D03G0041000	Cysteine proteinase inhibitor 8, Pollen allergen Phl p 5.0101	[21,26]
S5A_568799967	568.799967	Intergenic region	TraesCS5A03G0881300-TraesCS5A03G0882600	RuBisCO large subunit-binding protein subunit alpha, chloroplastic (Fragment), B2 protein	
S5A_569526776	569.526776	Intergenic region	TraesCS5A03G0882600-TraesCS5A03G0882900	B2 protein	[27]
S5A_570718644	570.718644	Intergenic region	TraesCS5A03G0885700-TraesCS5A03G0887200	Acyl-coenzyme A thioesterase 13	[20]
S5A_570788577	570.788577	Intergenic region	TraesCS5A03G0887200-TraesCS5A03G0887300	Acyl-coenzyme A thioesterase 13	[20]
S5A_594133493	594.133493	Intergenic region	TraesCS5A03G0949400-TraesCS5A03G0949800	Gibberellin-regulated protein 8, Snakin-1	[20]
S5A_698528417	698.528417	Intergenic region	TraesCS5A03G1257600-TraesCS5A03G1258800	CCR4-NOT transcription complex subunit 1	[22,28]
S5B_679675578	679.675578	Intergenic region	TraesCS5B03G1245900-TraesCS5B03G1246800	N/A	[21]
S5D_43408942	43.408942	Upstream gene variant	TraesCS5D03G0110300	BTB/POZ and MATH domain-containing protein 1	
S5D_486749507	486.749507	Intergenic region	TraesCS5D03G0937000-TraesCS5D03G0938500	N/A	[21,22]
S6B_562488824	562.488824	Intergenic region	TraesCS6B03G0886600-TraesCS6B03G0886800	Fatty acid desaturase DES2	[20]
S6B_601138481	601.138481	Intergenic region	TraesCS6B03G0954400-TraesCS6B03G0954500	Methyltransferase-like protein 7A	
S6B_610963068	610.963068	Intergenic region	TraesCS6B03G0966200-TraesCS6B03G0966500	Probable galacturonosyltransferase-like 9, Formin-like protein 16	[19]
S6B_610963076	610.963076	Intergenic region	TraesCS6B03G0966200-TraesCS6B03G0966500	Probable galacturonosyltransferase-like 9, Formin-like protein 16 (Bhatta et al., 2018b) [19]	[19]
S6D_27846508	27.846508	Intergenic region	TraesCS6D03G0114300-TraesCS6D03G0114400	60S ribosomal protein L13-1, AT-hook motif nuclear-localized protein 20	
S6D_29369738	29.369738	Intergenic region	TraesCS6D03G0114300-TraesCS6D03G0114400	60S ribosomal protein L13-1, AT-hook motif nuclear-localized protein 20	
S6D_376894590	376.89459	Intergenic region	TraesCS6D03G0607800-TraesCS6D03G0608400	Abscisic acid 8′-hydroxylase 1, WAT1-related protein	
S6D_454012454	454.012454	Intergenic region	TraesCS6D03G0764500-TraesCS6D03G0764700	Guanine nucleotide exchange factor subunit RIC1	[19]
S6D_469161928	469.161928	Intergenic region	TraesCS6D03G0810400-TraesCS6D03G0810500	N/A	[18,19,22]
S7B_723334278	723.334278	Intergenic region	TraesCS7B03G1219500-TraesCS7B03G1220400	N/A	[19,20,29]
S2A_726322626	726.322626	Intergenic region	TraesCS2A03G1140100-TraesCS2A03G1140200	VQ motif-containing protein 25	[30]
S2A_738732586	738.732586	Downstream gene variant	TraesCS2A03G1180900	Golgin candidate 2	[20,21,31]
S2A_751844369	751.844369	Intergenic region	TraesCS2A03G1227800-TraesCS2A03G1227900	N/A	[20]

* 5 Mb up-/downstream of MEA.

**Table 6:** Distribution of reference SNPs for elemental concentration in genetic diversity panel (GDP) of four germplasm groups.

Element	No. of SNPs	% of Reference SNP Alleles in Germplasm Groups:			
		Synthetics-CIMMYT	Synthetics-Japan	USA Cultivars	KASIB Germplasm
Frequency of germplasm		28.5	5.1	10.2	56.2
Ca	7	26.7	4.3	10.6	58.4
Mg	8	29.4	4.0	8.3	58.2
P	7	31.8	5.6	12.0	50.7
S	1	20.7	6.3	7.2	65.8
Cu	1	27.9	2.9	13.5	55.8
Mn	5	25.4	1.1	9.4	64.1
Cd	7	28.8	2.2	8.2	60.8
Ni	1	24.1	0.0	3.4	72.4
Mo	1	32.5	5.3	12.3	50.0
Sr	3	23.2	3.3	8.1	65.4
Mg, P	1	37.8	7.1	12.2	42.9
P, Zn	2	26.9	4.8	11.2	57.0
Cd, Mn	1	18.6	8.1	14.0	59.3
Cd, Mn, Zn	1	13.0	9.1	16.9	61.0

**Table 7:** Genotypes with optimal elemental concentration and their agronomic performance in 2017–2018.

Entry #	Genotype	Yield		Protein Content		1000 Kernel Weight		Optimal Concentration of the Following Elements *
		g/m^2^	+LC	%	+LC	g	+LC	
-	Pamyati Azieva, local check (LC)	399		16.5		43.5		-
12	Aisberg/*Ae. squarrosa* (511)	245	−38.7	16.8	2.3	43.4	−0.3	Ca, S, Cu, Fe, Mo
13	Ukr-Od 1530.94/*Ae. squarrosa* (392)	313	−21.7	17.2	4.2	42.0	−3.6	Mg, Fe, Mn, Rb, Sr
36	Aisberg/*Ae. squarrosa* (369)//Demir	273	−31.7	16.3	−0.7	47.5	9.1	Mg, Cu, Zn, Cd, Co, Mo
57	Ukr-Od 1530.94/*Ae. squarrosa* (392)	209	−47.7	18.2	10.7	47.6	9.4	K, P, S, Zn, Cd, Mo, Sr
14	Langdon/KU-2075	92	−77.1	21.1	28.1	40.3	−7.6	P, Cu, Fe, Mn, Cd, Rb
22	Langdon/IG 48042	141	−64.7	20.7	25.8	41.8	−4.1	Ca, Fe, Cd, Ni, Mo
47	Langdon/KU-2093	105	−73.8	21.5	30.5	47.0	7.8	K, Mg, S, Zn, Co, Sr
72	Tom	285	−28.7	19.2	16.7	40.0	−8.1	K, S, Cd, Mo, Sr
73	Freyr	354	−11.5	19.0	15.4	35.0	−19.6	P, Zn, Cd, Co, Ni, Mo
94	Element-22	535	33.8	17.0	3.4	44.7	2.7	Ca, P, S, Cu, Ni
96	Lutescens-96-12	432	8.1	16.8	2.2	44.0	1.0	Mg, Cu, Fe, Mo, Sr
99	Lutescens-6-04-4	486	21.8	18.1	9.7	47.3	8.6	K, S, Cu, Mn, Cd
103	Lutescens-15-12	372	−6.8	17.6	6.7	43.8	0.5	P, Cu, Co, Ni, Sr
114	OmGAU-90	466	16.7	15.7	−4.6	40.4	−7.2	Ca, Mg, S, Cu, Fe
116	Uralosibirskaya	515	28.9	17.6	6.6	49.5	13.6	K, Mg, S, Fe
119	Duet	427	6.8	16.0	−2.8	41.0	−5.8	Ca, S, Mn, Ni, Mo
128	GVK-2161	449	12.3	17.3	5.1	42.0	−3.6	Mg, Fe, Mn, Zn
132	Lutescens-248-01	394	−1.3	15.9	−3.3	49.8	14.2	Mn, Zn, Mo, Sr
136	Lutescens-48-204-03	369	−7.5	16.1	−2.1	49.0	12.6	P, Zn, Cd, Co, Ni, Mo
143	Lutescens-1103	469	17.5	16.1	−2.2	43.6	0.0	Ca, Mg, Mn, Ni, Mo
156	Novosibirskaya-41	482	20.6	19.0	15.3	39.7	−8.7	P, Cu, Cd, Co, Ni, Mo
157	OmGAU-100	518	29.7	16.4	−0.2	43.2	−0.9	Ca, P, Cu, Zn, Cd, Ni, Mo, Rb
164	Silach	541	35.4	16.7	1.2	49.8	14.3	Ca, Mg, Fe, Ni, Rb

* The optimal concentration was ranking in the 20% highest values for macro- and microelements and in 20% lowest values for trace elements based on average data for 2017–2018.

## Data Availability

The phenotypic data is available on request from the corresponding author.

## Supplementary Materials

The following are available online at https://www.mdpi.com/article/10.3390/plants11162173/s1, Table S1. ANOVA F-value probability of the effects of genotypes, year and their interaction for days to heading, grain yield, protein content, TKW and element composition for diversity panel. Table S2. Variation for agronomic traits and elemental grain concentration in genetic resources groups in 2017-2018. Table S3. SNPs with effects on grain elemental composition identified in GDP using GWAS. Table S4. SNPs with significant effect (*p* < 0.05) on single element concentration in wheat grain. Table S5. SNPs with significant effect (*p* < 0.05) on multiple elements concentration in wheat grain. Table S6. ANOVA F-value significance of the effects of genotypes, year, sites and their interaction for grain yield, protein content and elemental composition. Table S7. Distribution of reference SNPs for elemental concentration in genetic diversity panel (GDP) for four germplasm groups. Table S8. Agronomic performance and optimal concentration of elements in genetic diversity (GDP) based on mean values for 2017–2018. Table S9. Wheat genotypes of GWAS GDP used in the study. Table S10. The main soil parameters, air temperature and rainfall at experimental sites in 2017–2018 and over the long term (LT). Table S11. Correlations coefficients between agronomic traits and element concentrations in grain, 2017–2018.
