# Supplementary material for: Genetic Characterization of Spring Wheat Germplasm for Macro-, Microelements and Trace Metals
Source: Plants (Basel). 2022 Aug 21;11(16):2173. doi: 10.3390/plants11162173 (PMC9412593; doi:10.3390/plants11162173)
Supplement: Supplementary file 1 [file plants-11-02173-s001.zip › plants-1864911-supplementary.pdf]

Table S1. ANOVA F-value probability of the effects of genotypes, year and their interaction for days to heading, grain yield, protein content, TKW and element composition for diversity panel.

**Trait/Element**	**Genotypes**	**Year**	**Genotypes x year**	**H^2**
Days to heading	<0.001	<0.001	<0.001	0.84
Grain yield	<0.001	<0.001	<0.001	0.82
Protein content	<0.001	<0.001	<0.001	0.86
TKW	<0.001	1	<0.001	0.78
**Macroelements**				
Ca	<0.001	<0.001	<0.001	0.62
K	<0.001	0.48	<0.001	0.59
Mg	<0.001	0.18	<0.001	0.69
P	<0.001	<0.01	<0.001	0.58
S	<0.001	0.22	<0.001	0.62
**Microelements**				
Cu	<0.001	<0.01	<0.05	0.67
Fe	<0.001	0.15	1.00	0.47
Mn	<0.001	<0.05	<0.001	0.57
Zn	<0.001	<0.01	0.21	0.37
**Trace elements**				
Cd	<0.001	<0.01	0.21	0.67
Co	<0.001	<0.001	0.002	0.65
Ni	<0.001	>0.05	>0.05	0.49
Mo	<0.001	<0.05	0.20	0.82
Rb	0.12	<0.001	0.98	0.48
Sr	<0.001	>0.05	0.06	0.77

H^2 indicates broad-sense heritability.

Table S2. Variation for agronomic traits and elemental grain concentration in genetic resources groups in 2017-18.

Traits	Year	Germplasm groups					
		Pamyati Azieva	Synthetics - Mexico	Synthetics - Japan	USA	KASIB	All
No. of entries	-	1	37	8	14	64	135
Days to heading	2017	34.9±0.5	44.6±0.9	51.8±1.0	34.2±0.6	40.1±0.6	40.7±
	2018	36.4±0.4	41.6±0.5	42.5±0.5	35.2±0.4	39.0±0.4	40.2±
Grain yield, g/m^2	2017	378±5	217±13	82±15	255±10	407+6	322±10
	2018	425±9	255±9	125±10	385±14	491+9	394±11
Protein content, %	2017	17.0±0.1	17.7±0.15	21.3±0.22	18.5±0.17	17.2+0.13	17.7±0.12
	2018	16.0±0.1	17.6±0.14	20.4±0.11	17.2±0.16	16.2+0.11	17.0±0.12
TKW, g	2017	42.1±0.6	45.4±0.8	45.3±2.1	36.5±0.9	44.7+0.4	43.9±0.4
	2018	45.0±0.6	43.3±0.6	46.1±1.4	37.2±0.7	45.5+0.4	44.1±0.3
**Macroelements**							
Ca	2017*	378±9	372±9	379±28	380±14	350+6	362±5
	2018	392±8	407±9	428±15	424±11	378+6	394±5
K	2017	3869±39	3810±77	3753±91	3218±74	3605+36	3645±32
	2018*	3249±86	4039±86	4019±122	2936±129	3564+60	3629±49
Mg	2017	1185±13	1206±11	1189±23	1296±13	1202+7	1210±6
	2018	1255±11	1201±11	1176±23	1312±17	1227+7	1228±6
P	2017	5230±62	5077±49	5034±73	5051±59	5249+36	5170±25
	2018	4700±26	4657±61	4662±100	4723±63	4708+28	4693±23
S	2017	2091±23	2073±20	2211±32	1988±25	2052+14	2063±10
	2018	2141±19	2031±22	2179±39	1937±25	2061+13	2053±11
**Microelements**							
Cu	2017	4.86±0.05	4.73±0.09	4.73±0.18	4.57±0.19	4.63+0.05	4.67±0.04
	2018	4.04±0.09	3.77±0.09	3.58±0.11	3.64±0.15	3.70+0.04	3.73±0.04
Fe	2017	41.3±1.0	34.9±0.9	36.3±1.7	42.3±1.3	37.6+0.5	37.5±0.4
	2018*	36.4±1.3	36.8±0.7	38.8±2.1	39.4±1.3	34.2+0.5	35.8±0.4

Mn	2017	41.9±0.7	42.7±0.6	44.3±2.1	40.9±1.4	43.7+0.3	43.1±0.3
	2017	49.2±1.0	42.3±1.8	44.1±1.1	41.8±1.4	46.0+0.3	44.7±0.3
Zn	2017	53.8±2.1	52.5±0.8	53.5±3.2	57.4±1.7	53.1+0.6	53.3±0.5
	2018	44.1±0.8	42.2±1.1	38.4±2.5	41.5±1.2	42.0+0.6	41.9±0.5
**Trace elements**							
Cd	2017	0.050±0.003	0.039±0.002	0.069±0.007	0.041±0.002	0.043+0.001	0.044±0.001
	2018	0.033±0.002	0.026±0.001	0.047±0.006	0.031±0.002	0.032+0.001	0.031±0.001
Co	2017*	0.012±0.001	0.009±0.001	0.008±0.001	0.014±0.001	0.010+0.001	0.010±0.001
	2018*	0.010±0.001	0.009±0.001	0.010±0.001	0.009±0.001	0.009+0.001	0.009±0.001
Ni	2017	0.22±0.01	0.20±0.01	0.21±0.02	0.22±0.02	0.21+0.01	0.21±0.01
	2018	0.14±0.01	0.15±0.01	0.14±0.02	0.14±0.01	0.15+0.01	0.15±0.01
Mo	2017	0.34±0.01	0.35±0.01	0.37±0.02	0.31±0.02	0.34+0.01	0.34±0.01
	2018	0.33±0.01	0.29±0.01	0.34±0.01	0.29±0.01	0.32+0.01	0.31±0.01
Rb	2017*	4.32±0.27	4.77±0.18	5.32±0.43	3.56±0.15	4.29+0.09	4.37±0.08
	2018*	3.38±0.24	3.87±0.13	3.97±0.22	3.15±0.14	3.37+0.07	3.51±0.06
Sr	2017	1.91±0.09	2.35±0.10	2.19±0.28	2.16±0.16	1.95+0.07	2.09±0.05
	2018	2.35±0.07	2.62±0.09	2.47±0.19	2.51±0.20	2.31+0.06	2.42±0.05

*Original data

Table S4. SNPs with significant effect ($p < 0.05$) on single element concentration in wheat grain.

Element	Group 1: SNPs with significant effects in two years using original or adjusted values with $-\log_{10}(p\text{-value}) > 3.5$	Group 2: SNPs with significant effects in one year using original and adjusted values with $-\log10(p\text{-value}) > 4.0$
Ca	S3B_807804964*; S5D_43408942*; S6D_27846508*	S5A_569526776*; S5A_568799967*; S5A_570718644*; S5A_570788577*; S5A_698528417
K	-	S3A_717015583*; S7A_697829323*
Mg	S1B_468389275; S1B_470419196; S1B_483598145; S2A_738732586; S4B_64816370; S5A_464470946; S5B_679675578; S6D_469161928; S7B_723334278	S5B_684230407
P	S6B_610963068**; S6B_610963076; S6B_634042904*; S6D_376894590	S1D_17998333
S	-	S3B_57332301; S3B_727935439; S7A_630096604
Cu	S6D_29369738	S2B_103905079
Fe	-	S4A_677271805; S7B_595077897
Mn	S2A_24200649; S3A_697506434; S4B_603519569; S7B_574853540; S7B_720831474	-
Zn	-	S1B_639426265; S2B_114747112; S2D_22352746; S3D_604601826
Cd	S2A_751844369; S2B_772063522; S2B_780665986*; S2B_88259062; S2B_90578567*; S3D_550209436 S4D_11471805; S5D_486749507 S7B_677743542	S1A_443628214; S1B_553939861; S1B_553939884; S4A_629423563; S4A_632950380; S7A_262636; S7A_274842; S7A_3846016
Ni	S3B_758201335*	S1A_587375711; S2D_644205061; S3B_642865559; S3B_776544962; S3D_225856460; S3D_260458987; S3D_264592856; S3D_299071599; S3D_48835323; S3D_589420528; S3D_599387707; S4A_713385318; S5B_545403241; S5B_580595709; S6B_463558417; S6D_469583807
Mo	S2A_726322626*	S2A_642255440; S3A_596515072; S3B_769469095
Sr	S5A_698528417*; S6D_454012454	S2B_549201894; S5A_594133493

*SNP effects validated using KASIB multilocational trial. **SNP effects subjected to validation but not validated.

Table S5. SNPs with significant effect ($p < 0.05$) on multiple elements concentration in wheat grain.

Elements and year	SNPs with significant effects in two years using original or adjusted values with −log10(p-value) >3.5
Mg-17, P-17	S1A_118848466; S1A_41743658; S1B_10111796*; S1B_114437220*; S1B_13242483*; S1B_176291121*; S1B_184771090*; S1B_39706117; S1B_9711623*; S1D_106373551; S1D_34628004; S1D_47316263; S2A_3761912
Ca-17, S-17	S6D_100520815; S6D_129784877; S6D_138906498; S6D_147159935; S6D_151958101; S6D_158448100; S6D_16772839; S6D_178850661; S6D_218410466; S6D_229301846; S6D_286785478; S6D_290320292; S6D_300893611; S6D_332805923; S6D_342024645; S6D_377222506; S6D_380576720; S6D_389651664; S6D_413275134; S6D_420758890; S6D_441891592; S6D_445910207; S6D_52491545; S6D_53560537; S6D_55104815; S6D_7423323; S6D_79491780
Ca-17, S-17, Zn -17	S6D_14518544; S6D_458025359
Ca-17, K-17, P-18	S6D_4292779
Mg-17, P-17, S-18	S7B_23910463
Mg-17, Mo-18, P-17	S7D_418916815
Mg-17, P-17, Fe-17	S1A_85337461
P-17, S-17	S6B_452176632
P-18, Zn-18	S6B_37660171
P-17, S-17, Zn-17	S1A_576357185; S6B_562488824*; S6B_601138481*
Mo-17, P-18	S1B_555776959
Cd-18, Mn-17	S2B_780115106*
Cd-18, K-17	S7A_674019372
Mn-17, Zn-17	S2B_780665986*
Cd-18, Zn-17	S4A_706493915
Cd-17, S-17	S5A_32988364
Cu-17, Mn-17	S3A_728007201
Cu-17, Mn-17, P-17	S5A_685435772
Mn-18, S-17	S4B_23355392*
Mn-17, P-17	S5A_685435768
Mn-17, P-17, S-17, Zn-17	S5A_18798185
Ni-18, Zn-18	S4B_635994009
Ni-18, P-18, Zn-18	S7A_721773944

*SNP effects validated using KASIB multilocational trial.

Table S6. ANOVA F-value significance of the effects of genotypes, year, sites and their interaction for grain yield, protein content and elemental composition.

**Trait/ Element**	**F values significance level for effects and interactions:**						
	**Genotypes**	**Year**	**Site**	**Genotypes - year**	**Genotype - site**	**Year x site**	**Genotypes – year - site**
Grain yield	<0.001	<0.001	<0.001	<0.001	<0.001	<0.001	<0.001
Protein content	<0.001	<0.001	<0.001	<0.001	<0.001	<0.001	<0.001
Macro-elements							
Ca	<0.001	<0.001	<0.001	0.17	0.37	<0.001	<0.05
K	<0.001	<0.001	<0.001	0.10	0.17	<0.001	<0.001
Mg	<0.001	0.18	<0.001	0.37	0.70	<0.001	0.21
P	<0.001	<0.001	<0.001	0.19	0.45	<0.001	<0.001
S	<0.001	<0.001	<0.001	0.19	0.91	<0.001	0.17
Micro-elements							
Cu	<0.001	<0.001	<0.001	0.66	0.46	<0.001	<0.01
Fe	<0.001	<0.001	<0.001	0.05	0.25	<0.001	<0.001
Mn	<0.001	<0.001	<0.001	0.83	0.78	<0.001	0.23
Zn	<0.001	<0.001	<0.001	0.73	0.73	<0.001	0.34
Toxic trace elements							
Cd	<0.001	<0.05	<0.001	0.07	<0.01	<0.01	<0.01
Co	<0.001	<0.001	<0.001	0.49	<0.001	<0.001	<0.01
Ni	<0.001	<0.001	<0.001	0.52	<0.001	<0.001	<0.01
Trace elements							
Mo	<0.001	<0.001	<0.001	0.77	<0.05	<0.001	<0.05
Rb	<0.001	<0.001	0.35	0.66	0.14	<0.001	0.15
Sr	<0.001	<0.001	<0.001	0.19	<0.05	<0.001	<0.05

Table S7. Distribution of reference SNPs for elemental concentration in genetic diversity panel (GDP) for four germplasm groups.

SNP	Element	% of reference SNP alleles in germplasm groups:			
		Synthetics-CIMMYT	Synthetics-Japan	USA cultivars	KASIB germplasm
Frequency of germplasm		**28.5**	**5.1**	**10.2**	**56.2**
S3B_807804964	Ca	22.8	0.0	6.9	70.3
S5A_568799967	Ca	32.9	0.0	10.1	57.0
S5A_569526776	Ca	17.3	9.3	13.3	60.0
S5A_570718644	Ca	36.0	7.0	8.1	48.8
S5A_570788577	Ca	29.5	6.7	11.4	52.4
S5D_43408942	Ca	23.4	4.7	11.2	60.7
S6D_27846508	Ca	24.8	2.8	12.8	59.6
Average	**Ca**	**26.7**	**4.3**	**10.6**	**58.4**
S1B_468389275	Mg	36.6	5.9	13.9	43.6
S1B_470419196	Mg	36.6	5.9	13.9	43.6
S1B_483598145	Mg	31.5	6.5	1.9	60.2
S2A_738732586	Mg	28.4	0.0	8.6	62.9
S4B_64816370	Mg	27.0	0.9	4.5	67.6
S5B_679675578	Mg	29.8	5.8	4.1	60.3
S6D_469161928	Mg	23.8	1.0	8.9	66.3
S7B_723334278	Mg	21.8	5.9	10.9	61.3
Average	**Mg**	**29.4**	**4.0**	**8.3**	**58.2**
S1B_10111796	P	37.5	6.3	8.8	47.5
S1B_114437220	P	35.6	6.9	13.9	43.6
S1B_13242483	P	31.0	8.0	12.6	48.3
S1B_184771090	P	36.6	5.9	13.9	43.6
S1B_9711623	P	37.6	6.9	12.9	42.6
S6B_610963068	P	18.5	0.0	11.1	70.4
S6D_376894590	P	25.5	4.9	10.8	58.8

Average	**P**	**31.8**	**5.6**	**12.0**	**50.7**
S4B_23355392	**S**	**20.7**	**6.3**	**7.2**	**65.8**
S6D_29369738	**Cu**	**27.9**	**2.9**	**13.5**	**55.8**
S2A_24200649	Mn	29.8	0.0	8.3	62.0
S3A_697506434	Mn	27.2	5.6	8.0	59.2
S4B_603519569	Mn	23.1	0.0	12.0	65.0
S7B_574853540	Mn	17.1	0.0	9.8	73.2
S7B_720831474	Mn	30.1	0.0	8.9	61.0
Average	**Mn**	**25.4**	**1.1**	**9.4**	**64.1**
S2A_751844369	Cd	30.7	5.5	5.5	58.3
S2B_772063522	Cd	29.0	0.0	9.7	61.3
S2B_88259062	Cd	30.6	5.6	5.6	58.1
S3D_550209436	Cd	23.0	2.5	11.5	63.1
S4D_11471805	Cd	29.1	1.6	11.0	58.3
S5D_486749507	Cd	29.3	0.0	7.1	63.6
S7B_677743542	Cd	30.0	0.0	7.3	62.7
Average	**Cd**	**28.8**	**2.2**	**8.2**	**60.8**
S3B_758201335	**Ni**	**24.1**	**0.0**	**3.4**	**72.4**
S2A_726322626	**Mo**	**32.5**	**5.3**	**12.3**	**50.0**
S5A_594133493	Sr	35.6	5.8	12.5	46.2
S5A_698528417	Sr	10.5	0.0	0.0	89.5
S6D_454012454	Sr	23.5	4.2	11.8	60.5
Average	**Sr**	**23.2**	**3.3**	**8.1**	**65.4**
S1B_176291121	**Mg, P**	**37.8**	**7.1**	**12.2**	**42.9**
S6B_562488824	P, Zn	26.2	4.8	11.1	57.9
S6B_601138481	P, Zn	27.6	4.9	11.4	56.1
Average	**P, Zn**	**26.9**	**4.8**	**11.2**	**57.0**
S2B_780115106	**Cd, Mn**	**18.6**	**8.1**	**14.0**	**59.3**
S2B_780665986	**Cd, Mn, Zn**	**13.0**	**9.1**	**16.9**	**61.0**

Table S8. Agronomic performance and optimal concentration of elements in genetic diversity (GDP) based on mean values for 2017-18.

Entry #	Cross (Name)	Yield, g/m2	Yield, ±LC	PC, %	PC, ±LC	TKW, g	TKW, ±LC	Best 20% elements
MEAN	*Pamyati Azieva*	399		16.5		43.5		
12	AISBERG/AE.SQ.(511)	245	-38.7	16.8	2.3	43.4	-0.3	CaSCuFeMo
13	UKR-OD 1530.94/AE.SQ.(392)	313	-21.7	17.2	4.2	42.0	-3.6	MgFeMnRbSr
36	AISBERG/AE.SQ.(369)//DEMIR	273	-31.7	16.3	-0.7	47.5	9.1	MgCuZnCdCoMo
57	UKR-OD 1530.94/AE.SQ.(392)	209	-47.7	18.2	10.7	47.6	9.4	KPSZnCdMoSr
14	LANGDON/KU-2075	92	-77.1	21.1	28.1	40.3	-7.6	PCuFeMnCdRb
22	LANGDON/IG 48042	141	-64.7	20.7	25.8	41.8	-4.1	CaFeCdNiMo
47	LANGDON/KU-2093	105	-73.8	21.5	30.5	47.0	7.8	KMgSZnCoSr
72	Tom	285	-28.7	19.2	16.7	40.0	-8.1	KSCdMoSrPc
73	Freyr	354	-11.5	19.0	15.4	35.0	-19.6	PZnCdCoNiMo
94	Element 22	535	33.8	17.0	3.4	44.7	2.7	CaPSCuNi
96	Lut. 96-12	432	8.1	16.8	2.2	44.0	1.0	MgCuFeMoSr
99	Lut. 6/04-4	486	21.8	18.1	9.7	47.3	8.6	KSCuMnCd
103	Lut. 15-12	372	-6.8	17.6	6.7	43.8	0.5	PCuCoNiSr
114	OmGAU-90	466	16.7	15.7	-4.6	40.4	-7.2	CaMgSCuFe
116	Uralosibirskaya	515	28.9	17.6	6.6	49.5	13.6	KMgSFe
119	Duet	427	6.8	16.0	-2.8	41.0	-5.8	CaSMnNiMo
128	GVK 2161	449	12.3	17.3	5.1	42.0	-3.6	MgFeMnZn
132	Lutestsens 248/01	394	-1.3	15.9	-3.3	49.8	14.2	MnZnMoSr
136	Lutestsens 48-204-03	369	-7.5	16.1	-2.1	49.0	12.6	PZnCdCoNiMo
143	Lutestsens 1103	469	17.5	16.1	-2.2	43.6	0.0	CaMgMnNiMo
156	Novosibirskaya 41	482	20.6	19.0	15.3	39.7	-8.7	PCuCdCoNiMo
157	OmGAU-100	518	29.7	16.4	-0.2	43.2	-0.9	CaPCuZnCdNiMoRb
164	Silach	541	35.4	16.7	1.2	49.8	14.3	CaMgFeNiRb
MEAN	*Serebristaya*	471	17.9	14.9	-9.6	43.2	-0.9	
86	L 656	405	1.3	16.8	1.9	47.8	9.9	PMnRbSr
87	L 485	378	-5.4	17.0	3.5	43.6	0.0	CaPMnZn
102	Stolypinskaya 2	449	12.4	17.4	5.5	47.7	9.4	MgCoMoSr
115	Tyumenskaya Yubileynaya	381	-4.7	15.5	-5.8	41.1	-5.7	FeZn
117	Omskaya 36	475	18.9	15.7	-4.9	48.8	12.0	SFe
121	Novosibirskaya 18	477	19.4	15.4	-6.3	43.0	-1.3	
131	Lutestsens 932	339	-15.1	16.5	0.1	43.1	-1.1	KCuFe

137	Lutestsens 2028	417	4.4	16.0	-2.7	41.3	-5.2	MoSr
151	Lutestsens KS 963	572	43.2	17.2	4.6	40.0	-8.2	CaKMgCu
153	Lutestsens 1300	463	16.0	16.4	-0.3	42.5	-2.5	CaKCo
155	Novosibirskaya 16	390	-2.3	18.6	13.0	42.8	-1.8	MgSCdRb
159	Lut. 79/04-11	471	17.9	17.4	5.6	43.3	-0.6	MnCd
163	Tyumenochka	443	11.0	16.6	0.7	46.8	7.5	SZnNi
165	Eritrospermum 24841	453	13.4	16.9	2.4	42.0	-3.6	NiRb
97	Lut. 7/04-10	491	22.9	16.9	2.7	51.2	17.6	K
98	Lut. 186/04-61	487	21.8	18.5	12.5	48.8	12.1	KSr
108	Lut. 124-13	393	-1.6	18.1	9.9	45.3	3.9	SCdCoSr
109	Lut. 90-12	435	8.9	16.5	0.3	42.7	-2.0	PCoSr
111	Lut. 16-14	484	21.2	16.9	2.4	43.4	-0.4	FeMnZn
112	Lut. 15-14	557	39.4	16.5	0.2	42.6	-2.3	ZnRb
127	GVK 2127	399	-0.2	16.9	2.8	42.7	-1.9	MgMnMo
139	Lutestsens 30	457	14.4	15.9	-3.7	45.6	4.7	Co
144	Eritrospermum 1119	375	-6.2	16.6	1.0	44.8	2.9	CaPCdCo
146	Lutestsens 22-17	503	25.9	15.7	-4.7	51.4	18.0	SMoRb
148	Lutestsens KS 14/09-2	461	15.3	17.3	4.8	45.6	4.7	KNi
83	Lut. 79/04-3	471	17.8	16.7	1.6	50.2	15.2	P
84	Lut. 220/03-32	452	13.1	18.9	15.0	47.7	9.5	KPMnSr
85	Lut. 310-00-1	514	28.6	17.5	6.5	44.3	1.6	KPMnRb
89	Aestivum 947	514	28.6	15.8	-3.9	46.8	7.4	MgMnNiRb
91	Lut. 7/04-4	541	35.5	17.5	6.5	48.7	11.9	Zn
92	Lut. 242/97-2-21	499	25.0	17.2	4.4	47.6	9.4	PCoNiRb
93	Lut. 242/97-2-32	473	18.3	18.5	12.6	46.6	7.1	KPFeCd
95	Lut. 27-12	457	14.5	16.6	0.7	46.4	6.4	CuZn
101	LD-25	475	19.0	17.0	3.0	44.1	1.2	CaKCuCd
104	Lut. 87-12	414	3.7	17.5	6.6	42.2	-3.1	CuFeCdNi
105	Pamyati Leontyeva	467	16.8	16.0	-2.8	45.8	5.2	SFeCoMo
106	Lut. 88-13	482	20.7	17.2	4.7	51.8	19.0	CuRb
107	Lut. 23-12	416	4.2	17.2	4.5	43.3	-0.5	MgSCoMo
113	Pavlogradka	413	3.4	15.7	-4.6	48.8	12.1	Rb
118	Omskaya 35	511	27.9	16.3	-0.8	45.5	4.5	CaKSCd
122	Tobolskaya	457	14.3	15.3	-6.8	46.8	7.5	Cu
123	Sibirskiy Alyans	363	-9.2	16.6	1.1	47.8	9.8	SCdSr
124	Stepnaya 245	399	-0.1	16.4	-0.3	48.3	11.0	PNiMo
125	Stepnaya 253	468	17.2	14.1	-14.3	41.9	-3.8	CaMgFeZn
126	Stepnaya 259	461	15.4	15.5	-5.8	45.5	4.5	RbSr
129	Lutestsens 857	266	-33.3	15.6	-5.4	43.9	0.8	Sr
133	Lutestsens 393/05	386	-3.5	14.8	-10.0	39.4	-9.6	CoSr
134	Liniya 4-10-16	377	-5.7	18.4	11.8	40.7	-6.7	FeZn
135	Liniya 22 ChS	344	-13.9	17.6	7.0	49.3	13.1	Mo
138	Lutestsens 2102	375	-6.2	15.0	-9.1	44.4	1.9	MgMnSr
141	Lutestsens 65	391	-2.2	16.1	-2.2	41.6	-4.4	FeMnZn
142	Lutestsens 261	377	-5.6	15.8	-4.2	42.4	-2.7	Rb

145	Lutestsens 8-108-1	440	10.2	16.8	1.9	42.3	-2.8	MgCuCo
147	Lutestsens 37-17	502	25.5	16.0	-2.7	50.7	16.4	KZnYld
149	Lutestsens KS 140/08-3	459	14.8	18.0	9.3	46.3	6.4	
152	Lutestsens 1296	521	30.4	15.6	-5.3	41.8	-4.0	CaKCd
154	Sibirskaya 21	447	11.8	16.8	2.2	42.6	-2.1	CdRb
158	Lut. 3/04-21-11	512	28.1	17.2	4.3	49.2	13.0	Ca
161	SPChS 69	500	25.1	16.9	2.4	46.9	7.8	Co
162	Eritrospermum 59	490	22.7	16.0	-3.0	42.5	-2.3	S
5	LANGDON/KU-2096	74	-81.6	20.9	27.1	44.5	2.2	PZn
21	LANGDON/KU-20-9	120	-70.0	20.8	26.2	48.8	12.0	CuMoSr
51	LANGDON/IG 131606	97	-75.7	20.6	25.1	43.8	0.6	CaCoNi
63	LANGDON/KU-2092	93	-76.7	21.0	27.8	52.5	20.6	MgRb
65	LANGDON/KU-2105	108	-73.1	20.2	22.8	46.8	7.5	KSMn
3	UKR-OD 952.92/AE.SQ.(1031)	255	-36.2	17.0	3.3	43.8	0.6	ZnCoRbSr
4	UKR-OD 1530.94/AE.SQ.(310)	225	-43.7	18.5	12.5	43.0	-1.2	PCu
6	UKR-OD 1530.94/AE.SQ.(458)	213	-46.6	17.7	7.6	42.8	-1.7	PCdCo
7	UKR-OD 952.92/AE.SQ.(1031)	276	-30.9	17.8	8.0	41.8	-4.1	Ca
9	UKR-OD 952.92/AE.SQ.(1031)	209	-47.8	18.2	10.7	47.2	8.3	PZnSr
16	UKR-OD 1530.94/AE.SQ.(458)	206	-48.5	17.2	4.3	44.2	1.6	CaFeCdRb
18	AISBERG/AE.SQ.(369)	312	-21.9	18.3	10.8	45.5	4.4	
19	UKR-OD 1530.94/AE.SQ.(1027)	227	-43.1	16.8	1.9	36.8	-15.4	KCu
23	AISBERG/AE.SQ.(511)	243	-39.1	18.3	11.0	47.5	9.0	CaSMnMo
24	AISBERG/AE.SQ.(511)	219	-45.2	19.2	16.9	51.2	17.6	MgSFeMn
25	UKR-OD 1530.94/AE.SQ.(458)	243	-39.2	16.4	-0.3	40.0	-8.1	KCu
26	PANDUR/AE.SQ.(223)	121	-69.8	17.3	5.0	40.6	-6.8	FeCdMo
27	LEUC 84693/AE.SQ.(409)	234	-41.5	18.0	9.1	47.0	8.0	CaS
28	AISBERG/AE.SQ.(369)	199	-50.1	18.1	9.8	42.8	-1.7	Mn
31	AISBERG/AE.SQ.(369)	228	-43.0	17.9	8.7	45.1	3.5	MgMnCo
32	AISBERG/AE.SQ.(369)	306	-23.4	16.8	1.8	42.1	-3.3	MgCdMoRb
33	UKR-OD 1530.94/AE.SQ.(629)	166	-58.4	18.5	12.2	41.5	-4.7	Cu
35	UKR-OD 1530.94/AE.SQ.(629)	147	-63.3	18.5	12.5	38.4	-11.9	KPSr
37	UKR-OD 1530.94/AE.SQ.(310)	234	-41.5	18.6	12.8	47.3	8.6	MgNi
38	AISBERG/AE.SQ.(369)	243	-39.1	17.9	8.6	44.1	1.3	MgFeZnCd
41	UKR-OD 1530.94/AE.SQ.(1027)	175	-56.3	19.8	19.9	42.8	-1.8	PNi

42	UKR-OD 1530.94/AE.SQ.(310)	241	-39.7	16.9	2.9	40.2	-7.6	
43	UKR-OD 952.92/AE.SQ.(1031)	278	-30.3	16.9	2.3	43.0	-1.2	Fe
44	UKR-OD 1530.94/AE.SQ.(1027)	298	-25.4	16.9	2.4	48.2	10.7	CoNi
45	UKR-OD 1530.94/AE.SQ.(1027)	261	-34.6	17.7	7.5	41.9	-3.9	CaMnNiRb
46	UKR-OD 1530.94/AE.SQ.(1027)	268	-32.9	17.7	7.4	54.8	25.7	ZnNiSr
48	UKR-OD 1530.94/AE.SQ.(1027)	165	-58.8	17.5	6.4	43.7	0.2	Zn
49	UKR-OD 1530.94/AE.SQ.(1027)	177	-55.6	17.4	5.7	43.2	-0.9	KNi
55	AISBERG/AE.SQ.(511)	251	-37.2	17.0	3.4	49.4	13.4	CaMoRb
56	UKR-OD 1530.94/AE.SQ.(1027)	264	-34.0	18.2	10.7	45.2	3.9	KSCoNi
59	UKR-OD 1530.94/AE.SQ.(1027)	248	-37.9	17.0	3.3	45.5	4.4	MnRbSr
61	PANDUR/AE.SQ.(409)	330	-17.5	17.4	5.7	46.1	5.9	KSCu
62	UKR-OD 1530.94/AE.SQ.(1027)	229	-42.8	17.1	3.7	44.0	1.0	PCo
66	RBOT	386	-3.3	18.4	11.8	35.0	-19.7	PSCu
68	Linkert	286	-28.3	19.2	16.5	39.7	-8.9	KMg
69	Rollag	276	-31.0	18.6	13.0	37.5	-13.9	CuFeRb
71	Sadin	356	-11.0	18.8	14.2	35.6	-18.4	CoMo
74	Knudson	270	-32.3	18.0	9.2	38.7	-11.1	CaS
75	Kelby	286	-28.5	19.1	16.0	33.8	-22.4	MgCuMnSr
76	Jenna	350	-12.3	19.0	15.5	43.3	-0.6	CdCoNi
77	Brennan	297	-25.7	18.7	13.9	32.4	-25.7	MgFeMnSr
78	Alpine	321	-19.7	17.9	8.8	37.6	-13.6	PZnRb
79	SY TYRA	305	-23.6	18.3	11.0	33.9	-22.2	CaZn
81	Prevail	390	-2.3	18.3	11.1	37.1	-14.7	FeMn
82	Advance	321	-19.6	17.4	5.5	36.8	-15.5	CaKNiRb

Table S9. Wheat genotypes of GWAS GDP used in the study.

**Entry #**	**Cross (Name)**	**Country of origin**	**Institution**
**CHECKS**			
160	*Pamyati Azieva*	Russia	Omsk AC
150	*Serebristaya*	Russia	Omsk AC
**SYNT-MEXICO**			
3	UKR-OD 952.92/AE.SQUARROSA(1031)	MX-TCI*	CIMMYT
4	UKR-OD 1530.94/AE.SQUARROSA(310)	MX-TCI	CIMMYT
6	UKR-OD 1530.94/AE.SQUARROSA(458)	MX-TCI	CIMMYT
7	UKR-OD 952.92/AE.SQUARROSA(1031)	MX-TCI	CIMMYT
9	UKR-OD 952.92/AE.SQUARROSA(1031)	MX-TCI	CIMMYT
12	AISBERG/AE.SQUARROSA(511)	MX-TCI	CIMMYT
13	UKR-OD 1530.94/AE.SQUARROSA(392)	MX-TCI	CIMMYT
16	UKR-OD 1530.94/AE.SQUARROSA(458)	MX-TCI	CIMMYT
18	AISBERG/AE.SQUARROSA(369)	MX-TCI	CIMMYT
19	UKR-OD 1530.94/AE.SQUARROSA(1027)	MX-TCI	CIMMYT
23	AISBERG/AE.SQUARROSA(511)	MX-TCI	CIMMYT
24	AISBERG/AE.SQUARROSA(511)	MX-TCI	CIMMYT
25	UKR-OD 1530.94/AE.SQUARROSA(458)	MX-TCI	CIMMYT
26	PANDUR/AE.SQUARROSA(223)	MX-TCI	CIMMYT
27	LEUC 84693/AE.SQUARROSA(409)	MX-TCI	CIMMYT
28	AISBERG/AE.SQUARROSA(369)	MX-TCI	CIMMYT
31	AISBERG/AE.SQUARROSA(369)	MX-TCI	CIMMYT
32	AISBERG/AE.SQUARROSA(369)	MX-TCI	CIMMYT
33	UKR-OD 1530.94/AE.SQUARROSA(629)	MX-TCI	CIMMYT
35	UKR-OD 1530.94/AE.SQUARROSA(629)	MX-TCI	CIMMYT
36	AISBERG/AE.SQUARROSA(369)//DEMIR	TCI	CIMMYT
37	UKR-OD 1530.94/AE.SQUARROSA(310)	MX-TCI	CIMMYT
38	AISBERG/AE.SQUARROSA(369)	MX-TCI	CIMMYT
41	UKR-OD 1530.94/AE.SQUARROSA(1027)	MX-TCI	CIMMYT

42	UKR-OD 1530.94/AE.SQUARROSA(310)	MX-TCI	CIMMYT
43	UKR-OD 952.92/AE.SQUARROSA(1031)	MX-TCI	CIMMYT
44	UKR-OD 1530.94/AE.SQUARROSA(1027)	MX-TCI	CIMMYT
45	UKR-OD 1530.94/AE.SQUARROSA(1027)	MX-TCI	CIMMYT
46	UKR-OD 1530.94/AE.SQUARROSA(1027)	MX-TCI	CIMMYT
48	UKR-OD 1530.94/AE.SQUARROSA(1027)	MX-TCI	CIMMYT
49	UKR-OD 1530.94/AE.SQUARROSA(1027)	MX-TCI	CIMMYT
55	AISBERG/AE.SQUARROSA(511)	MX-TCI	CIMMYT
56	UKR-OD 1530.94/AE.SQUARROSA(1027)	MX-TCI	CIMMYT
57	UKR-OD 1530.94/AE.SQUARROSA(392)	MX-TCI	CIMMYT
59	UKR-OD 1530.94/AE.SQUARROSA(1027)	MX-TCI	CIMMYT
61	PANDUR/AE.SQUARROSA(409)	MX-TCI	CIMMYT
62	UKR-OD 1530.94/AE.SQUARROSA(1027)	MX-TCI	CIMMYT
**SYNT-JAPAN**			
5	LANGDON/KU-2096	Japan	Kyoto Univ.
14	LANGDON/KU-2075	Japan	Kyoto Univ.
21	LANGDON/KU-20-9	Japan	Kyoto Univ.
22	LANGDON/IG 48042	Japan	Kyoto Univ.
47	LANGDON/KU-2093	Japan	Kyoto Univ.
51	LANGDON/IG 131606	Japan	Kyoto Univ.
63	LANGDON/KU-2092	Japan	Kyoto Univ.
65	LANGDON/KU-2105	Japan	Kyoto Univ.
**USA**			
66	Rbot	USA	Univ. of Minnesota
68	Linkert	USA	Univ. of Minnesota
69	Rollag	USA	Univ. of Minnesota
71	Sadin	USA	Univ. of Minnesota
72	Tom	USA	Univ. of Minnesota
73	Freyr	USA	Univ. of Minnesota
74	Knudson	USA	Univ. of Minnesota

75	Kelby	USA	Univ. of Minnesota
76	Jenna	USA	Univ. of Minnesota
77	Brennan	USA	Univ. of Minnesota
78	Alpine	USA	Univ. of Minnesota
79	SY Tyra	USA	Syngenta
81	Prevail	USA	Univ. of Minnesota
82	Advance	USA	Univ. of Minnesota
**KASIB**			
165	Eritrospermum 24841**	Russia	Chelyabinsk ARI
137	Lutestsens 2028	Kazakhstan	Karagandy ARI
131	Lutestsens 932**	Kazakhstan	KIZ
151	Lutestsens KS 963**	Russia	Kurgan Seed
117	Omskaya 36	Russia	Omsk AC
159	Lutestsens 79-04-11**	Russia	Omsk AC
102	Stolypinskaya 2**	Russia	Omsk SAU
153	Lutestsens 1300	Russia	Samara ARI
155	Novosibirskaya 16**	Russia	Siberian ARI
156	Novosibirskaya 41**	Russia	Siberian ARI
121	Novosibirskaya 18	Russia	Siberian ARI
87	L 485	Russia	South-East ARI
86	L 656	Russia	South-East ARI
115	Tyumenskaya Yubileynaya**	Russia	Tyumen SAU
163	Tyumenochka**	Russia	Tyumen SAU
124	Stepnaya 245**	Kazakhstan	Aktobe AES
126	Stepnaya 259**	Kazakhstan	Aktobe AES
125	Stepnaya 253**	Kazakhstan	Aktobe AES
143	Lutestsens 1103**	Russia	Altay ARI
123	Sibirskiy Alyans	Russia	Altay ARI
122	Tobolskaya	Russia	Altay ARI
135	Liniya 22 ChS**	Kazakhstan	Karabalyk AES

136	Lutestsens 48-204-03**	Kazakhstan	Karabalyk AES
134	Liniya 4-10-16**	Kazakhstan	Karabalyk AES
138	Lutestsens 2102**	Kazakhstan	Karagandy ARI
133	Lutestsens 393-05**	Kazakhstan	Kaz. GRI
132	Lutestsens 248-01**	Kazakhstan	Kaz. GRI
129	Lutestsens 857**	Kazakhstan	KIZ
145	Lutestsens 8-108-1**	Russia	Kurgan ARI
147	Lutestsens 37-17**	Russia	Kurgan ARI
149	Lutestsens KS 140-08-3**	Russia	Kurgan Seed
161	SPChS 69**	Russia	Omsk AC
85	Lutestsens 310-00-1	Russia	Omsk AC
91	Lutestsens 7-04-4	Russia	Omsk AC
118	Omskaya 35**	Russia	Omsk AC
92	Lutestsens 242-97-2-21	Russia	Omsk AC
158	Lutestsens 3-04-21-11**	Russia	Omsk AC
83	Lutestsens 79-04-3	Russia	Omsk AC
93	Lutestsens 242-97-2-32	Russia	Omsk AC
116	Uralosibirskaya	Russia	Omsk AC
84	Lutestsens 220-03-32	Russia	Omsk AC
114	OmGAU-90	Russia	Omsk SAU
113	Pavlogradka	Russia	Omsk SAU
119	Duet	Russia	Omsk SAU
104	Lutestsens 87-12	Russia	Omsk SAU
95	Lutestsens 27-12	Russia	Omsk SAU
106	Lutestsens 88-13	Russia	Omsk SAU
105	Pamyati Leontyeva	Russia	Omsk SAU
107	Lutestsens 23-12	Russia	Omsk SAU
162	Eritrospermum 59	Russia	Omsk SAU
103	Lutestsens 15-12	Russia	Omsk SAU
141	Lutestsens 65**	Kazakhstan	Pavlodar ARI

142	Lutestsens 261**	Kazakhstan	Pavlodar ARI
152	Lutestsens 1296**	Russia	Samara ARI
154	Sibirskaya 21**	Russia	Siberian ARI
101	LD-25	Russia	South-East ARI
89	Aestivum 947	Russia	
144	Eritrospermum 1119	Russia	Altay ARI
164	Silach**	Russia	Chelyabinsk ARI
127	GVK 2127**	Kazakhstan	East-Kaz, ARI
128	GVK 2161**	Kazakhstan	East-Kaz, ARI
146	Lutestsens 22-17**	Russia	Kurgan ARI
148	Lutestsens KS 14-09-2**	Russia	Kurgan Seed
98	Lutestsens 186-04-61	Russia	Omsk AC
99	Lutestsens 6-04-4	Russia	Omsk AC
97	Lutestsens 7-04-10	Russia	Omsk AC
157	OmGAU-100**	Russia	Omsk SAU
109	Lutestsens 90-12	Russia	Omsk SAU
94	Element 22**	Russia	Omsk SAU
96	Lutestsens 96-12	Russia	Omsk SAU
108	Lutestsens 124-13	Russia	Omsk SAU
112	Lutestsens 15-14	Russia	Omsk SAU
111	Lutestsens 16-14	Russia	Omsk SAU
139	Lutestsens 30**	Kazakhstan	Pavlodar ARI

*MEX-TCI – Mexico and Turkey-CIMMYT-ICARDA program

**Genotypes included in KASIB validation trial

Table S10. The main soil parameters, air temperature and rainfall at experimental sites in 2017-2018 and over the long term (LT).

Site	Humus, %	pH	Total content, μg/g⁻			Temperature May-August, °C			Rainfall May-August, mm		
			$N\text{-}NO_3$	P_2O_5	K_2O	2017	2018	LT	2017	2018	LT
Karabalyk, KZ	4.5	7.0	0.010	0.090	0.210	18.7	17.6	17.9	209	197	186
Shortandy, KZ	3.5	7.7	0.008	0.124	0.420	18.0	15.3	17.0	104	244	168
Chelyabinsk, RU	5.2	5.3	0.005	0.117	0.110	16.9	16.1	15.0	243	313	231
Omsk, RU	5.0	6.8	0.010	0.130	0.196	17.4	15.2	16.4	141	231	207
Novosibirsk, RU	7.5	6.7	0.012	0.131	0.070	16.8	15.3	15.9	271	249	220
Tyumen, RU	7.0	6.7	0.008	0.160	0.180	15.5	14.8	15.8	283	307	249

Table S11. Correlations coefficients between agronomic traits and element concentrations in grain, 2017-2018.

Element	Year	Yield	PC	TKW	Ca	K	Mg	P	S
Ca	2017	-0.11	0.06	-0.14	-	0.16	0.15	0.01	0.09
	2018	-0.14	0.06	-0.15	-	-0.03	0.22*	0.03	0.11
K	2017	-0.13	0.03	0.10	0.16	-	-0.07	-0.07	0.30*
	2018	-0.19	0.16	0.17	-0.03	-	0.07	0.07	0.09
Mg	2017	-0.24*	0.43*	-0.07	0.15	-0.07	-	0.79*	0.60*
	2018	-0.19	0.29*	-0.06	0.22*	0.07	-	0.76*	0.55*
P	2017	-0.14	0.32*	0.08	0.01	0.20*	0.79*	-	0.68*
	2018	-0.22*	0.30*	0.05	0.03	0.48*	0.76*	-	0.20*
S	2017	-0.23*	0.45*	0.13	0.09	0.30*	0.60*	0.68*	-
	2018	-0.34*	0.47*	0.12	0.11	0.09	0.55*	0.20*	-
Cu	2017	-0.14	0.28*	-0.02	-0.03	0.10	0.62*	0.68*	0.54*
	2018	-0.21*	0.25*	0.01	0.08	0.21*	0.60*	0.60*	0.47*
Fe	2017	-0.10	0.23*	0.29*	-0.01	-0.14	0.23*	-0.06	-0.09
	2018	-0.13	0.17	-0.14	-0.03	-0.12	0.05	-0.04	-0.10
Mn	2017	-0.13	0.35*	-0.02	0.11	-0.10	0.68*	0.57*	0.49*
	2018	-0.09	0.05	0.09	0.22*	-0.10	0.63*	0.46*	0.38*
Zn	2017	-0.03	0.20*	0.04	-0.08	0.02	0.53*	0.73*	0.48*
	2018	-0.30*	0.26*	0.02	0.09	0.27*	0.49*	0.63*	0.50*
Cd	2017	-0.20*	0.29*	0.01	0.00	-0.02	0.46*	0.44*	0.49*
	2018	-0.22*	0.38*	0.10	0.09	0.05	0.37*	0.32*	0.40*
Co	2017	0.01	0.07	-0.19	0.12	0.14	0.17	0.14	0.04
	2018	0.01	0.05	0.04	0.19	0.12	0.11	0.15	0.15
Ni	2017	-0.08	0.15	-0.01	-0.01	0.09	0.25*	0.25*	0.23*
	2018	-0.24*	0.36*	0.01	-0.03	0.14	0.23*	0.28*	0.31*
Mo	2017	-0.07	0.20*	0.05	0.01	0.17	0.12	0.20*	0.24*
	2018	-0.02	0.15	0.18	0.05	0.27*	0.21*	0.33*	0.26*
Rb	2017	-0.05	0.06	0.16	-0.02	0.12	0.04	0.08	0.07
	2018	-0.18	0.12	0.01	0.07	0.18	-0.01	0.00	0.16

Sr	2017	-0.34*	0.22*	-0,08	0.60*	0.11	0.17	-0.00	0.16
	2018	-0.34*	0.31*	-0.22*	0.65*	0.04	0.17	0.04	0.16
Number of significant correlations		11	18	3	4	6	16	14	15

*, **, ***significant at $p > 0.05$, 0.01 and 0.001, respectively.
